# Modifications of Textile Materials with Functional Silanes, Liquid Silicone Softeners, and Silicone Rubbers—A Review

**DOI:** 10.3390/polym14204382

**Published:** 2022-10-17

**Authors:** Jerzy J. Chruściel

**Affiliations:** ŁUKASIEWICZ Research Network—Lodz Institute of Technology, Brzezińska Str. 5/15, 92-103 Łódź, Poland; jerzy.chrusciel@lit.lukasiewicz.gov.pl

**Keywords:** fibers, textile materials, cellulose, cotton, polyesters, auxiliary chemical substances, functional silanes, polysiloxanes (silicones), silicone softeners, silicone oils, silicone elastomers and rubbers, superhydrophobic properties

## Abstract

General information concerning different kinds of chemical additives used in the textile industry has been described in this paper. The properties and applications of organofunctional silanes and polysiloxanes (silicones) for chemical and physical modifications of textile materials have been reviewed, with a focus on silicone softeners, silane, and silicones-based superhydrophobic finishes and coatings on textiles composed of silicone elastomers and rubbers. The properties of textile materials modified with silanes and silicones and their practical and potential applications, mainly in the textile industry, have been discussed.

## 1. Introduction

Textile materials are composed of different natural polymers (mainly cellulosic fibers from cotton or modified cotton), synthetic polymers [usually polyesters (mainly polyethylene terephthalate (PET)), polyacrylates (PAA), and their copolymers, but also other polymers and copolymers] and polymer blends (most often mixtures of cotton and PET fibers with different weight ratios). About 33% of cotton is still used for the production of textiles in the world. Synthetic fibers: polyester, polyamide, polypropylene, polyethylene-*co*-vinyl acetate (EVA), polyurethane elastomer (spandex, Lycra, elastane), (polyvinyl alcohol) (PVA), or other highly plastic fibers account for over 60% of consumption [1].

The modification processes of the properties of natural or synthetic fibers and textile goods with different groups of chemical additives have quite a long history (see Table 1 for some examples). Fabric softeners (conditioners) are applied in washing machines in order to reduce harshness in clothes (or other textile goods) during drying in air after machine washing. The additives of fabric softeners play the role of after-treatment laundry aid, in contrast to laundry detergents. Moreover, very useful are additions of more dilute preparations of wrinkle releasers which are sprayed directly onto fabrics [2]. Surfactants and polyglycol products are used in textile processing as emulsifiers in order to improve the wetting and penetrating properties of textile materials. Softeners are the next important and very large group of auxiliary chemical compounds used for textiles treatment because they reduce the friction coefficient between fibers leading to the surface softness and lubricating effect on fibers. They are applied in order to impart softness and improve the wear feeling of textile goods [3]. Many kinds of softeners are used on a large scale in the final finishing of textiles and clothes. There is practically no product available today for clothing or home use that has not been treated with the softener. In this way, the correct behavior of the fiber product during the packaging process is conditioned or its grip is corrected, making it more functional and more pleasant to use. The action of textile softeners is usually based on the oriented adsorption of the active substance on the fiber surface. Standard organic softeners contain hydrophobic fatty residues with a hydrocarbon chain length of C_16_ to C_18_. These substances are deposited in an orderly manner on the surface of the fibers. Thus, the individual fibers move against each other much easier, and the softened product gives the impression of being decidedly softer than a non-softened textile product.

The main purpose of using softeners is:

- an improvement in the aesthetic quality of the product, which is usually organo-leptically described as soft, smooth, fluffy, and with a more flexible grip,
- a positive effect on such technological features of the product as antistatic, moisture absorption, flexibility, an improvement of sewing, wipeability, a reduction in the tendency to pilling, etc.,
- for products composed of synthetic fibers, it is possible to correct the grip, making it similar to natural fibers [4].

The softeners are not only used for the final finish of the product but also to improve or obtain the right effect in such processes as scratching, sanforization, sewing, and yarn rewinding [4].

The softeners for textiles should have the following characteristics [3,4,5,6,7]:

1. ease of use (e.g., solution stable after dilution),
2. good compatibility and miscibility with other additives used in finishing baths,
3. the simple finishing process,
4. gives good softness,
5. is skin-friendly—during treatment, no harmful gases are to be released and they cannot have an allergic or stimulating effect on the human body,
6. does not cause yellowing (and to turn yellow itself) or discoloration, which does not affect the resistance of dyes to sunlight,
7. they should not change the color of the staining,
8. low-foam and resistant to shear forces,
9. should not deposit on the surface of the padding machine rollers,
10. have good or very good affinity for agents used in batch methods to the fiber,
11. possibility of spray application,
12. be non-toxic and anticorrosive,
13. be easily biodegradable,
14. does not require special restrictions for road transport and storage (flash point),
15. good stability during high-temperature treatment in dyeing and finishing (e.g., non-volatile, not with water vapor),
16. they should be stable during storage, biodegradable, and meet the requirements of environmental protection
17. good washing resistance,
18. a moderate price.

Virtually none of the products available on the market meet all of the above expectations. Therefore, from the wide range of available resources, in order to give the products the expected properties, the softeners should be selected taking into account the conditions of use existing in the plant [4,8].

**Figure 1:**
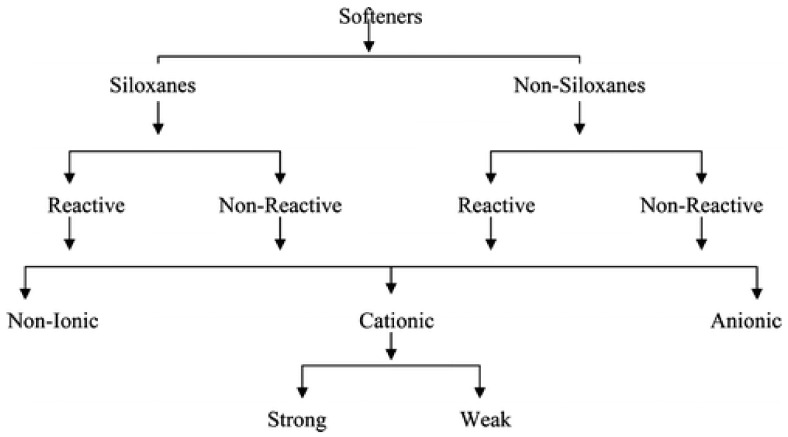
General classification of softeners [9]. Adapted with permission—Copyright American Chemical Society (2008).

Many kinds of softeners are applied in the textile industry: cationic, anionic, non-ionic based on paraffin and polyethylene, ethoxylated non-ionic, and silicone-based (Figure 1) [9,10]. Softeners are most often in the form of soft flakes or oil. Multiple ethyleneamines were often used as a raw material to prepare soft flakes or oils [11]. Depending on the type of amine used and its amount in relation to the acid used, condensation products of a non-ionic or cationic nature can be obtained. Products with a quaternary ammonium group were obtained by an additional amine quaternization process [4].

Non-ionic softeners were obtained in the process of the condensation of fatty amino acids with ethylene oxide. With some exceptions, non-silicone softeners are a group of compounds based on fatty amino acids (stearic C_17_H_35_COOH or palmitic C_15_H_31_COOH) [4,12]. Non-ionic softeners have the general chemical formula R(OC_2_H_4_)_n_OH, where R = alkyl group. They contain many kinds of non-ionic active ingredients: fatty alcohols, ethoxylated fatty alcohols and amines, paraffins, or oxidized polyethylene waxes, and can be easily blended with other active agents. They are thermally resistant and hardly turn yellow. These compounds are ideal for optically finishing brightened white fabrics or knitted fabrics [4,8]. Emulsions of polyethylene softeners in a water bath are stable due to the presence of alkaline additives. A typical composition of such a composition is as follows: polyethylene wax 20%, emulsifier 5%, KOH 0.5%, and water 74.5% [4].

Among the classic agents, cationic softeners are distinguished by the best softening of the textile material and relatively good durability in washing. Their softening properties result from the structure based on high-molecular fatty acids in combination with amines. They are soluble in water and also show antistatic properties, with a predominance of hydrophobic properties and good distribution over fiber, and even antibacterial effects. They are characterized by very good smoothing properties and, due to their substantivity to fibers, they are useful for almost all types of textiles, especially those composed of cellulose fibers, giving good results with very small concentrations. However, they can reduce the fastness to light of direct and reactive dyes and increase the susceptibility to soiling [4,13,14].

The cationic softeners are prepared from condensation reactions of aliphatic amines with fatty acids or esters. The following groups were described in the literature:

- amino functional softeners (primary fatty amines of the general formula R–CH_2_–NH_2_ or secondary fatty amines of the formula R–CH_2_–NH–CH_2_–R), which in an acidic environment form quaternary salts;
- fatty diamines R–NH–CH_2_–CH_2_–CH_2_–NH_2_;
- fatty aminoesters (reaction products of fatty alcohols with fatty acids) with long alkyl chain substituents (R) contain one or more amino groups—in an acidic environment, they show a cationic character proportional to the number of amino groups in the molecule, but their ester groups are not very resistant to hydrolysis in alkaline media;
- fatty aminoamides (condensation products of polyamines with fatty acids); they exist in the form of acetate, chloride, and sulfate salts;
- imidazoles, which exhibit lower viscosity than the amines from which they were obtained and, therefore, have a better softening effect on the textile product; they cause yellowing to a lesser extent than their starting products;
- quaternary ammonium salts [4].

Amphoteric softeners (quaternary amines and betaine derivatives) give textiles less softening but increase the antistatic properties and hydrophilicity of the products. They are often used in hygiene products because they are friendly to human skin. Their mixtures with amphoteric compounds are often used to finish velvet towels [4,8]. Commercial compositions of such compounds are recommended for special purposes. The softening effect is not always satisfactory, but due to their ionic nature, they can be used with caution in the same bath with anionic agents, e.g., optical brighteners. They stand out positively by improving the antistatic properties of the product and do not worsen the wettability. Moreover, this group of compounds has no negative effect on human skin and is mostly used to soften hygiene products and towels. Most of them are biodegradable products [4]. 

Anionic softeners are characterized by a negative charge of the molecules containing carboxyl (–COO^−^), sulfone (–OSO_3_^−^) or phosphate (–PO_4_^–^) groups. There are different types of these compounds:

- sulfonated fatty alcohols, e.g., cetyl sulfate in the form of sodium salt,
- sulfonated fatty acid amides or esters, e.g., the condensate of stearoyl chloride with the sodium salt of acid N-methylaminoacetic acid (sarcosine).

Anionic auxiliary agents require an appropriate sulfonation process or the incorporation of phosphate groups into the fatty compound [12].

To a certain degree, due to their chemical structure, softeners are similar to surfactants. Almost all surfactants can cause less or better softening of the textile, but not all commercial softeners can be detergents. The softeners for textiles are used as water emulsions containing 15% to 25% of an active product. Currently, apart from the group of classic compounds obtained in the condensation processes of the aliphatic derivatives of fatty hydrocarbons, multifunctional silanes and silicones form a newer group of softeners that is gaining more and more use, especially in the recent two decades. Silicones are also used as additives to other softeners. Moreover, different functional silanes and silicone rubbers are also often applied as modifiers of textiles properties [4,13,15].

Antimicrobial modifications of textile materials [16], and their protection against UV radiation with different kinds of modifiers [17], were also very often applied.

**Table 1:** A brief history of the development of additives applied in the technology of textile materials.

Period	Description of Innovations	References
1906–1909	- crosslinking of rayon with formaldehyde	[13,18,19]
1930s	- chemical modifications of products composed of natural and regenerated cellulose fibers in order to further improve their properties, especially their behavior during and after washing;	
	- elaboration of finishing agents reducing shrinkage and the crushing of regenerated cellulose fiber products with the use of urea-formaldehyde precondensation products;	[13,20,21,22,23]
1940s and 1950s	- application of melamine-urea derivatives was introduced into textile finishes, especially for modifications of artificial viscose fibers	[13,24,25,26,27,28,29,30]
1950–1990	- elaboration and application of cationic, anionic, non-ionic based on paraffin and polyethylene, ethoxylated non-ionic, silicone-based (Figure 1) [9,10,12,13,14] multiple ethyleneamines [11], amphoteric softeners (quaternary amines and betaine derivatives), and quaternary ammonium derivatives [4,8]; non-ionic softener (which was obtained in the process of condensation of fatty amino acids with ethylene oxide, mainly from fatty amino acids, based on stearic C_17_H_35_COOH or palmitic acid C_15_H_31_COOH [4,12]) and non-ionic softeners with the general chemical formula R(OC_2_H_4_)nOH, where R = alkyl group; they also contained different kinds of non-ionic components: fatty alcohols, ethoxylated fatty alcohols, ethoxylated fatty amines, paraffins, or oxidized polyethylene waxes [4,8];	[4,8,9,10,11,12,13,14]
	- application of the compositions of chemical compounds with high reactivity with the OH groups of cellulose were developed, e.g., methylol and alkoxymethyl compounds of cyclic urea derivatives, triazones, and compounds for the so-called non-resinous finishes, e.g., aldehydes and cyclic oxymethylenes or sulfone derivatives of betaine and epichlorohydrin;	[13,31,32,33,34,35,36,37,38,39,40,41]
1950 and 1960s	**The first generation of silicone auxiliaries** for textiles (very lubricating, low-cost PDMS oils, and telechelic hydroxyl-terminated PDMS) was introduced to the industry. They have also the advantages of the reduced color of the fabric after treatment but have poor washing resistance, the softness of the hand feeling, and poor rebound effect, and their emulsions can be unstable and stratified, resulting in the generation of silicone oil spots. The first-generation silicone softeners are very easy to demulsify and bleach the oil during use. At present, the first-generation products are rarely used as fabric softeners and generally are used as yarn lubricants, filler fiber treatment agents, or chemical fiber spinning finishes.	[9,13,15,42]
1980s	Since the 1980s was developed **the second generation of silicone softeners**, which includes epoxy or/and amino-modified polysiloxanes. Both groups are non-hydrophilic and easy to demulsify. The epoxy modification provides dryness while amino modification gives smoothness. They greatly improve the washing durability of the fabric. Amino-modified silicone oil gives a smooth feel and rebound effect, good washing resistance, and can improve the tearing strength of fabrics. However, it also has the disadvantages of the instability and delamination of the emulsion (leading to the generation of silicone oil spots), and also, very small yellowing and color change problems appear. Currently, amino-silicone oils are still the most often used modified silicone fabric softener on the market. Epoxy-modified silicone oil feels dry, has a rebound effect, and can improve the tearing strength of the fabric after treatment. However, after finishing, the fabric lacks fullness and softness, and due to the instability and delamination of the emulsion, the generation of silicone oil spots was observed. The water-absorbent fabric is not hydrophilic after finishing. Thus, it is mainly used in water-repellent soft finishing. In the production process, emulsion polymerization and the emulsification of silicone oil are carried out at the same time, the production cost is low, the emulsion particles are larger, and the smoothness is very good. The main disadvantage is that the softness of the hand feeling is not good, and there will be a small amount of precipitate during the storage of the product. It is mainly used in the smooth-feeling treatment of various fiber fabrics.	[42]
1990s	In the 1990s, **the third generation of polyether-modified silicone softeners**, containing polyether active groups grafted on the side chains of polysiloxane chains, was elaborated. They do not easily break the emulsion, and after finishing, give good hydrophilic properties to the fabrics, which are slightly worse when touching. They also include amino (epoxy)/polyether-modified silicone oils. The pure polyether-modified silicone oil has water-dispersible/water-soluble characteristics without emulsification, delamination, and oil spots, and the fabric can be re-dyed after treatment, it can provide instant water absorption function, and the fabric has no yellowing and color change after treatment. However, the finished fabric has poor smoothness, no plump and soft feeling, the hydrophilicity and softness are not durable, and the tearing strength of the fabric is not improved. This type of product is only suitable for those that need instantaneous water absorption and require general soft finishing. In comparison with monopolyether-modified silicone oil, amino (epoxy)/polyether-modified silicone oil has a better hand feeling than mono-polyether-modified silicone oil and slightly higher yellowing. It is mainly used as a general-purpose hydrophilic soft finishing for general fabric agents.	[42]
~2000	Around 2000 **the fourth generation of silicone softeners** was developed—linear multiblock polysiloxane copolymers, exhibiting good hydrophilicity, a soft feeling, low yellowing, and being difficult to break. The fourth-generation silicone softeners have the advantages of the first three generations of products and excellent comprehensive performance. It is very easy to disperse in water and it is convenient and simple to use them without any emulsifiers or with a small amount of emulsifiers. High-concentration products can be used directly, showing very good compatibility, excellent stability to strong acids, strong bases, high electrolytes, etc., very good high temperature and high shear stability, and extremely high adaptability and flexibility to processes and equipment. They find many practical applications for modifications of various fibers and fabrics. This kind of silicone softener uses no demulsification, does not stick to rollers and to cylinders, has good washing resistance, leaves no oil spots and very low yellowing and discoloration, and also provides a full, fluffy, and silky texture. It gives a natural comfort feeling, which overcomes the shortcomings of traditional amino silicone oil which is rather “too greasy”. It has a very good feel and can be super soft. It has medium or higher hydrophilicity, which can be the same as organic fluorine’s easy decontamination and finishing. It enables color repair, peelability, re-dyeing and over-dyeing, and no secondary pollution; it basically does not affect the heat migration fastness of dispersed dyes on polyester and maintains the color fastness to washing and rubbing before soft finishing.	[42]
2000–2022	Further progress in the development and many examples of applications of functional silanes and silicone-based softeners for the surface modifications of different kinds of fibers and textile materials is presented in the course of this publication.	

## 2. Chemical Structures of Silicone-Based Additives and Their Uses in the Textile Industry

Alternating silicon and oxygen (siloxane) bonds form a framework of silicon chains, with organic substituents attached to the silicon. Most often methyl groups exist as organic substituents in commercial silicones, most of which are polydimethylsiloxanes (PDMS) [43,44]. Due to their hybrid “inorganic-organic” chemical structure and the flexibility of the siloxane bonds, silicones have some unique properties, such as low-temperature flowability, high compressibility, low viscosity change vs. temperature, low surface tension (spreadability), thermal and oxidative stability, hydrophobicity, good dielectric properties, and low fire hazard [45].

Silicones show hydrophobic properties with low glass transition temperature, elasticity, low surface tension, and film forming ability. Their chain flexibility results from the rotational freedom of the Si–O–Si linkages and the low interaction energies between the methyl groups [46]. The silicone softeners exhibit better softening properties because of the lower rotational free energy of Si–O bonds than C–O bonds [3]. Chemically modified silicones with aminoalkyl groups attached to the PDMS backbone give the so-called ”supersoft” handle [46,47,48].

The surface energy and the surface tension of silicone oils are much lower than for mineral oils and organic liquids and result from their molecular structures, molecular weight, and viscosity of silicone oil. The surface tension of methylsilicone oils depends on temperature and viscosity: it has values of 18.7 mN/m at a viscosity of 2 mPa·s, 20.1 mN/m at a viscosity of 10 mPa·s, and 21.5 mN/m at higher viscosities. The surface tension of methyl(hydro)silicone oils is 20 mN/m. For methyl(trifluorophenyl)silicone oils, it is in a range of 20–22 mN/m and depends on the content of the fluoroalkyl substituents. The surface tension of methyl(phenyl)silicone oils is higher: 23–28 mN/m and it depends on the content of the phenyl groups. Low values of the surface tension of silicone oils affect their properties: the wettability of organic surfaces, hydrophobicity, and antiadhesive properties [49].

Silicones include oils and greases, emulsions, rubber products, and resins and they find applications in many diverse markets, such as electronics, construction, aerospace, automotive, medical materials, performance chemicals and coatings, personal care, paper goods, and textiles [50,51]. Silicones have broad utility in textile processing and finishing. Most of the products for the textile industry are based on functional silanes, polydimethylsiloxanes (PDMS), and their derivatives [52,53,54].

Silicones have been frequently applied in the textiles industry as process aids (e.g., lubricants), elastomeric finishes, coatings, and performance enhancers (as softeners and water repellents) [10], as fabric finishes for coatings, print paste softeners, and antifoam agents for fabric and carpet dyeing. Silicone antifoaming agents are often used in washing, dyeing, and bleaching processes. Silicones also serve as fiber lubricants for spinning, winding, and slashing, as wetting agents, water repellents, and softeners. Silicone thread lubricants meet the demands of high-speed industrial sewing machinery. Silicones are also often applied in nonwoven modifications, e.g., as binder additives for wet-laid processes [11,52].

**Figure 2:**
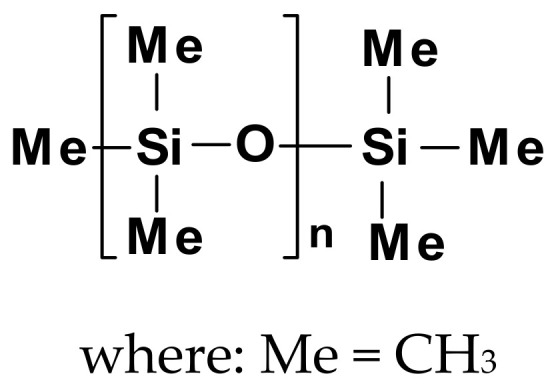
Chemical structure of the nonreactive methylsilicone oil.

Emulsions of nonreactive polydimethylsiloxanes (PDMS) (see a general structure in Figure 2) or amino-functional PDMS derivatives are the most often used silicones for modifications of textile properties. Emulsions and nanoemulsions of polysiloxanes with pendant aminoalkyl or *quaternary ammonium groups* (QAGs) are excellent softeners of textile materials.

**Figure 3:**
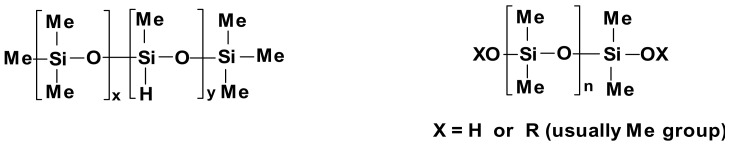
Chemical structures of reactive polysiloxanes [polymethylhydrosiloxanes (PMHS), and telechelic polydimethylsiloxane-α.ω-diols or α,ω-dialkoxy-PDMS).

**Figure 4:**
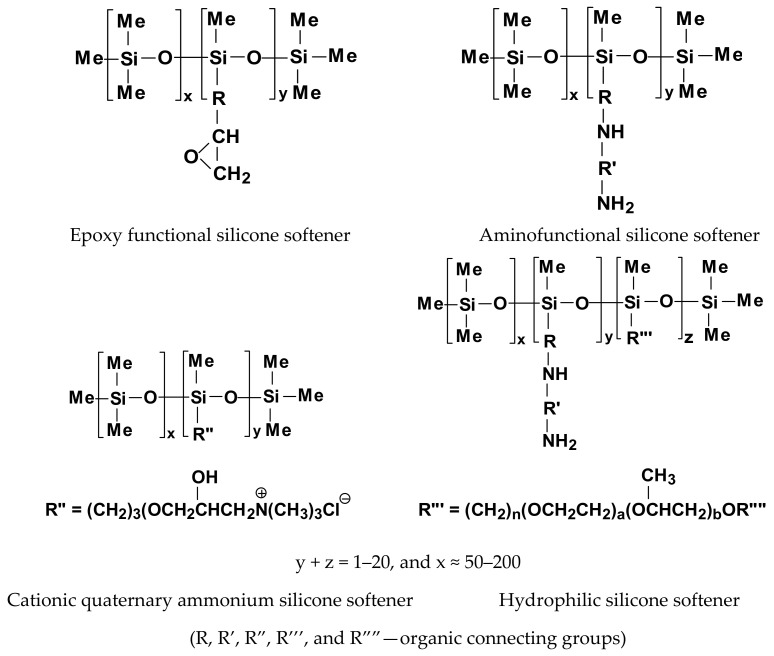
Examples of chemical structures of typical silicone softeners used in the textile industry.

Silicone additives and finishes enhance the performance of different modified materials and can be either nonreactive (Figure 2), reactive with silanol (Si–OH), (alkoxy)silane (Si–OR), or Si–H groups (Figure 3), or organofunctional silicones, containing different side or end groups: e.g., –NH_2_, –CH–CH_2_–O–(epoxy), –NR_3_^+^Cl^−^, –COOH, –NHCOR’, and –O(CH_2_CH_2_O)_a_[CH(CH_3_)O]_b_X (X = H or R) (like in Figure 4). Some silicones are used as neat fluids, others in the form of emulsions or as room temperature crosslinking elastomers. PDMSs are extremely versatile and are applied to formulate a wide range of compositions with tailored hydrophobicity and durability. They allow for modifying the feel and appearance of fabrics, improving processing, or achieving more than one benefit from a single product. Many kinds of silicone substrates show effectiveness even at very low concentrations. Thus, they provide the desired properties, which can improve the cost efficiency of textiles technology and help ensure a minimum environmental impact [52].

Silicone polyethers, i.e., comb-like polysiloxanes containing grafted segments of ether oligomers, silicone elastomers (SEs), and silane coupling and crosslinking agents (SCA) are also commonly used [55,56]. Examples of chemical structures of non-ionic and cationic silicone softeners, most often used in the textile industry, are presented in Figure 3 and Figure 4 [10].

The application area of silicone softeners in the textile industry depends on their molecular structures [57].

By means of the conventional measurement of the tensile strength method, the efficacy of an important group of silicone plasticizers (aminosilicones with different structures) was reliably distinguished. The following influencing parameters were differentiated: the type and the content of the amino groups, the emulsion, and the composition of the textile base (cotton or cotton/polyester fabric). The types of silicones tested were aminoethylaminopropyl silicones, cyclohexyl-aminopropyl silicones, and PDMS oil. A comparison of force/elongation plots in different elongation directions showed that a comparable deformation in the weft or diagonal direction requires significantly less force than in the warp direction. Aminoethylaminopropyl silicones performed less well than comparable comb polymers with the same amine number. The fabrics finished with comb aminosilicones were much more hydrophilic. Cyclohexyl-aminosilicones gave emollient effects that were comparable to those of aminoethyl-aminopropyl polysiloxanes but were slightly weaker for small numbers of amine groups but have pronounced hydrophilic properties. So far, the most important factor influencing the softening effect of silicones has been the content of amino groups. The basic tendency of the softness versus texture plots was essentially similar for both fabrics. A clear difference was the shape of softness in relation to the function of the amine number. The softening effect of aminopolysiloxane emulsions of various structures on various substrates (knitted fabrics, pure cotton, polyester, and their mixtures) was studied by the determination of elongation and shear hysteresis. Structure/effect relationships were defined and optimal values for the most important contents of amino groups. For pure cotton, a strong dependence on the amine number was determined, while polyester fabric showed a weaker relationship. Particle size and viscosity played a relatively minor role [44,58].

The adsorption kinetic of the commercial aminosilicone additive on knitwear and terry cotton was a concentration-dependent and kinetically controlled fast process. Within the first 200 s, 80% of the total amount of aminosilicone was adsorbed in a 2 µm-thick layer on knitwear and terry cotton [59].

In recent decades, silicones are the most often used softeners in the textile industry [60]. Amino-functional polysiloxanes and silicones containing polyether groups (very often ethylenoxy or propylenoxy) are the most commonly applied softeners for textiles. Modified silicone agents are used more and more often, ensuring, apart from the softening effects, also a certain hydrophilicity of the finished products. This effect is achieved by introducing amino groups into the side chain and results in a high softness and smoothing property [4]. Telechelic carbofunctional polysiloxanes containing terminal amino or hydroxy groups, apart from softness and smooth grip, give the products flexibility and trouble-free sewing of the fabrics and a reduction in creasing combined with very good durability of the washing and cleaning effects. Silicone agents containing *quaternary ammonium groups* in the molecule show high resistance to the action of alkalies and their emulsions of shear forces [61]. Silicone softeners produce an extraordinary degree of softness, flexibility, and elegance, and perform much more effectively than other softeners. The features given to textiles are directly related to the very high flexibility of the silicone polymer chain in combination with the low surface tension, which results in an extraordinary film-forming capacity and highly limited friction between the fibers [62,63]. Methylsiloxane or dimethylsiloxane copolymers and silicones containing ether segments or amino groups or carboxyl or amide or epoxy groups, allow to obtain the intended aesthetic values and optimal grip of the finished product [64].

Aminofunctional silicones give textiles, especially knitted fabrics, the final aesthetic effect. They allow to obtain the best product grip when used in a weak acid bath; they behave like quaternary salts creating strong interactions with the negative zeta potential surface of the cellulose fiber. A disadvantage of aminosilicone softeners is that the finished product tends to turn slightly yellow [4].

Formulations for textile finishing are typically used as silicone oil emulsions in water with a visually different degree of light scattering. Micro- (<0.01 μm) and macro-emulsions (<0.1 μm) have a milky appearance, while nano-emulsions are transparent [65,66]. The fabric’s inner structure is more easily penetrated by nano-silicone softeners than by others. The industrial applications of nano-silicone softeners increase rapidly. Modification with the nano-silicone softener did not have a significant effect on the color fastness properties of the knitted fabrics. Rubbing, abrasion and pilling resistance, dry cleaning, and washing fastness using four different knitted samples of silicone-modified textile materials were rated. Fabrics modified with the nano-silicone softeners showed poor abrasion but better pilling resistance, but they did not have a significant effect on the color fastness properties of the knitted fabrics [60].

Appropriately selected commercial emulsifiers allow to easily penetrate into single fibers inside the textile product, ensuring excellent internal softness, grip, and smoothness of the textile materials. Aminosilicone emulsions are sensitive to changes in bath pH. In an acidic environment, the amino groups, after attaching a proton, create the *quaternary* *ammonium group* that determines the stability of the emulsion. In an alkaline bath, the ammonium groups lose a proton, which makes the silicone less polar, and the stability of the emulsion decreases. Functional silicone softeners provide specific surface properties on various raw materials. The fluorescence spectroscopy and the flow potential analyses indicated that the transfer of silicone softeners from the emulsion to the textile surface was significantly better at a lower pH. It was proposed that a multi-layered silicone agent may be created on the fiber surface [66]. The softeners may affect the physical structure of the textile material composed of synthetic fibers—the hydrophobic part of the softening agent may penetrate the fiber, changing its arrangement, which results in a lower glass transition temperature of the fiber material [4].

The wide applications of softeners lead to a soft touch and a nice smell of clothes. Water-based formulations of household conditioners are very often based on *quaternary ammonium surfactants,* assembled in vesicles. Finally, these technologies were combined and novel industrial formulations containing two kinds of additives, surfactant molecules and silicone oil, were developed. Namely, the silicone oil modified with amino groups was added into a developed softener composed of vesicles of an esterquat and guar polymers [65]. Esterquats are a new generation of fabric softening agents containing quaternary ammonium groups, two long [C(16)-C(18)] fatty acid chains, and two weak ester linkages, serving as substitutes for dialkyl(dimethyl)-ammonium salts [67]. Esterquat or guar gum were useful as stabilizers for the incorporation of silicone softener into the fabric. The stability of the softener compositions based on silicone oils was studied by dynamic light scattering (DLS), cryogenic transmission electron microscopy (cryo-TEM), and optical and fluorescent microscopy. The silicone oil was stabilized by the surfactant and the guar gum in nano- or micro-metric droplets. The structure and properties of vesicles were not affected by the silicone oil, and the formulations were stable. An addition of silicone oil into a topical conditioner allowed the development of multi-purpose fabric softeners [65]. Obviously, the softeners with a *quaternary ammonium group* exhibited bacteriostatic properties. Effective bacterial removal was found already in a bath with detergent at levels of 81.3% and 89.8% for cotton and microfiber towels, respectively. On the other hand, the use of the softening agent resulted in a bacteria removal rate of 94.9% for a cotton towel and 72.0% for a microfiber towel [4,68]. The silicone softeners include dimethylsiloxane oils, comb-like polymethylsiloxanes containing polyether side segments, and liquid carbofunctional hydroxyl- or amino-terminated polydimethylsiloxanes. They are some of the most important after-treatment agents which play the role of emulsifiers and provide a significantly differentiated fabric feel. Silicone-based finishing and softening agents do not foam too much and show high emulsification efficiency (i.e., they easily form microemulsions), good wetting properties, and a good hand feeling of the fabric finish [69]. They have many useful features and benefits leading to:

- excellent fiber-to-fiber lubrication,
- good stretch and recovery,
- improved elasticity and resiliency,
- excellent surface protection,
- a “premium”, soft hand feeling,
- hydrophobic or hydrophilic properties (depending on the type),
- good durability.

The silicone softeners containing amino groups cause improved stability towards alkalies, excellent high-shear stability, and durable press bath and anionic compatibility, and also only low or medium yellowing (depending on the type) of the fabric finish [69].

Silicone finishes help to retain the shape of fabrics, texture, and abrasion resistance, and to achieve uniformity and brilliance of color. Silicones enable new techniques to design lightweight, durable, water-repellent, and high-performing sportswear, as well as to allow the fabric to keep air permeability (so-called “*breathability*”). Moreover, silicones are used in dry cleaning because they help to carry detergent while rinsing away suspended dirt and oils trapped by the detergent, therefore preserving the good quality and color of cleaned clothes. Modifications with silicones give soft textiles, even after repeated washings [69].

Both kinds of silicone softeners (non-ionic and cationic) provide high lubricity, very high softness, unique hand feeling, elastic resilience, crease recovery, abrasion resistance, tear strength, and good sewability. They show good temperature stability and durability, with a high degree of permanence for products forming crosslinked films and a range of properties from hydrophobic to hydrophilic [13,70]. Their very low glass transition temperatures (about −100 to −120 °C) and special elasticity result from the high molecular flexibility of the silicone chain [71,72]. The methyl groups of the dimethylsiloxane segments –[OSi(CH_3_)_2_]_n_– to a great extent shield the oxygen atoms from outside contact, making the surface of the fibers finished with PDMSs mostly non-polar and hydrophobic. Strong hydrogen bonds, which exist between the hydroxyl or amino groups of the fibers (in cellulose, wool, silk, and polyamide fibers) and the amino groups of the modified silicone, act as an anchor for the silicone, which forms an evenly distributed film on the fiber surface. It results in a very soft hand feeling and good water repellency. The polysiloxane segments between the anchor sites are long enough to keep their high flexibility when an optimal content of amino side groups is retained. It provides the softness and the lubricating effect of aminofunctional silicones on polar fibers. The hydrophobic surface of non-polar polyester fibers interacts strongly with the hydrophobic segments of the silicone chains. The positively charged amino side groups of the silicone chains repel each other and give rise to the enhanced flexibility of the silicone chain loops. This additionally results in the especially soft hand feeling of aminofunctional silicones on non-polar fibers [73]. Quaternary-modified groups also cause high shear and alkaline stability [10,74].

Good surface activity, a self-emulsifying property of modified polyester fabrics was achieved by the application of a series of amphiphilic polysiloxanes containing multicationic groups, which were prepared by the copolymerization of (octamethyl)cyclo-tetrasiloxane (Me_2_SiO)_4_ with *N*-(dimethylaminopropyl)-*N′*-aminopropyl(dimethoxy)-silane, followed by quaternization with benzyl chloride. They also exhibited better wettability than another commercial aminofunctional polysiloxane softener, AEAP-PDMS. These novel amphiphilic polysiloxanes containing multicationic groups should find many practical applications; e.g., they improve the wettability of composite biomaterials [75].

Silicone softeners also influenced the thermal decomposition and flammability of polyethylene terephthalate (PET). Nano- and micro-emulsions affect the thermal behavior of PET in terms of crystallinity degree, T_g_, T_m_, enthalpy, the initiation of the thermal degradation, and the weight of the residue at high temperatures. In the presence of silicone softener, PET decomposed at lower temperatures, and this process was highly affected by the content of the softeners. All coated polyester fabrics also had significantly higher flammability compared with non-treated fabrics, and the loading of applied softener onto fibers significantly affected their combustion properties. Nano- and micro-emulsion formed on the PET surface a three-dimensional scaffold, which decreased the surface free energy. It caused faster combustion by the pyrolysis, and the silicone-treated textile was further completely decomposed, giving a char [76].

The benefits of silicone additives and a wide range of their advantages for the modifications of textiles result from their low concentrations. Methyl silicones were inert and did not show a negative environmental effect. The advantages of the applications of silicone agents based on PDMSs in the technology of textile fabrics and nonwovens led to numerous replacements of organic materials, excellent implementation, and minimal environmental concerns. PDMS materials do not emit significant amounts of formaldehyde at typical process temperatures and do not form hazardous dioxins or furans during textile processing or waste treatment. A potential risk is further minimized because the average temperatures during textile processing are usually lower than 215 °C, and very often do not exceed 175–200 °C. The testing of PDMSs showed no evidence of toxicity to aquatic, terrestrial, or avian life forms, either plant or animal. Silicone additives are considered environmentally friendly alternatives for many other industrial chemicals applied in textile technology. The low contents and eco-safety of silicones indicate the likelihood of their brilliant future in the textile industry [53].

The amino-functionalized silicone oil additives have also found wide applications in the finishing (and dyeing) of white knit and woven cotton fabrics. Textiles with soft, fluffy properties were obtained. The whiteness, color strength, flexibility, fabric softness, crease recovery, absorbency, tensile strength, and abrasion resistance were increased after modification, together with excellent fastness properties. Silicone oil showed an excellent affinity to all kinds of fabrics used: knit and woven cotton fabrics, jute, etc. [77].

The emulsion of amino-modified vinyl telechelic polysiloxane surfactant was applied to the cotton fabrics. The hydrophilic property of finished cotton fabric was good. It had little effect on the whiteness property of finished cotton fabrics but had no effect on the shade of dyed finished fabric. It also had little effect on the strength of finished fabric, and the feel retention rate was 60% after several times of washing, which showed that the amino-modified silicone oil had a good wash fastness property. The rating of the hand feeling properties (softness and smoothness) of the finished cotton knit fabric was 5 and cotton woven was 4.5 on a scale of 1–8. The thermal stability of this emulsion (with a solid content of 25%, a viscosity of 60 mPa·s, and a pH of 6–7) and the dielectric stability were good at 90 °C, while the alkali stability was good at 80 °C [78].

Silicone oils and emulsions were used, not only as textile finishes but also as fiber lubricants and process supports, both for textiles in clothing and industry. Silicone elastomers and rubbers were used similarly to adhesives, binding additives, fabric coatings, dress lining, and thread sealers. The overall challenge for textiles treated with silicon materials was the enhancement of their properties. Treatment with silicone compounds led to new important properties of cellulosic fabrics. Silicone-modified textiles offered long-lasting comfort and efficiency. For example, currently produced jeans offer users the impression of utter warmth and shape. The use of silicones in textiles still has much potential for development since new silicone compounds or their mixtures can improve the functional properties of textile materials. Future applications of silicone-treated textiles in the therapeutic medical field are also possible [46].

The amino-functional silicone softener was better than the cationic and non-ionic softeners for finishing knitted cotton fabrics. Bleached and dyed fabrics created from Siro yarns showed better water-related comfort properties after treatment with 1% silicone softener. However, at a silicone softener content > 1%, a decrease in the properties of the treated fabrics was observed, probably due to the silicone double-layer formation, which could change the interaction of water with fabric [79].

Many factors affect the performance of finished knitted cotton fabrics with the hydrophilic amino- and polyether-silicone softeners, such as the content of the emulsifier, the chemical structure of the silicone softener, the choice of silicone coupling agent (SCA), and the mass ratio of the polyether silicones to the SCA. When emulsifying amino silicone oil softeners with water, only small additions of other emulsifiers provide good permeability. An insufficient amount of emulsifier, during the synthesis of polyether-silicone softeners from PMHS and polyether, will affect the amount of graft hydrophilic groups and the emulsifying effect on the products, resulting in a decrease in the hydrophilicity, antistatic property, emulsion stability, and handling of the softeners [3].

Emulsions of silicone-based softeners containing additives of glycerin, PEG 400 and PEG 4000 were utilized for the coating of the knitted cotton fabrics. A thermal comfort and handle and drape properties were enhanced. The performance properties of the cotton-knitted fabrics were also improved by the addition of silicone softeners. However, crucial changes in the mechanical and whiteness properties of the silicone-applied fabrics were not observed, and the hydrophilicity, the transfer or dispersion of the moisture, and the air permeability were even worsened. In order to obtain well-maintained, breathable, and easy-drying cotton surfaces, the use of softeners from the group of quaternary ammonium silicone oil, together with appropriate additives, should be preferred [80,81].

## 3. Modification of Surface Properties of Fibers and Textiles with Functional Silanes (and Silicones)

Sol–gel processes, utilizing different kinds of organofunctional silane coupling agents (SCA), have been especially useful for the modification of the surface properties of different textile materials, composed either of natural or synthetic fibers. Cotton fabrics composed of natural cellulosic fibers are often applied for the fabrication of hydrophilic and very hygienic textile materials; for instance, the surface modification of cellulosic fibers was carried out with an SCA in an ethanol/water medium. The silane functional groups participate in the chain growth and form the covalent bonds of a polymer matrix with the cellulosic fibers. Through the condensation of alkoxysilane groups with cellulose hydroxyl groups, their hydrolysis into silanol groups and the self-condensation between the Si–OH groups Si–O–Cellulose and Si–O–Si linkages were formed, which was confirmed by diffuse reflectance infrared spectroscopy. The change in the surface properties after the modification was studied by contact angle measurements and inverse gas chromatographic analysis [82].

Tetra(ethoxy)silane Si(OC_2_H_5_)_4_ (TEOS) and many different carbofunctional silanes were often used for multifunctional and nanocoating finishes in numerous modifications of textile materials properties by the sol–gel method, which has found many applications in the textile industry in the recent 15–20 years. In sol–gel technology, the inorganic metal salts or metal alkoxides have been applied, imparting high, durable activity and multifunctional properties to different textile materials, in a one-pot manner, using a low concentration of precursors in the same bath as an alternative to the so far applied textile finishing treatments [83,84,85,86].

B. Mahltig et al. substantially contributed to the development of the modification and functionalization of textile materials and the improvement of their properties with SCAs by sol–gel methods. Chemically or physically modified silica “nanosols”, with particle diameters smaller than 50 nm, were used for the coating of textile materials, leading to different changes in their physico-mechanical, optical, electrical, and biological properties. This way, the protection of the textiles against destruction and the creation of new expected and advantageous functions can be reached, and textiles characterized by:

- improved dyeing,
- increased abrasion,
- decreased inflammation,
- electrical conductivity,
- UV protection,
- water, oil, and soil repellency,
- controlled release of oil and flavor,

as well with biocompatible, biocatalytic, and antimicrobial properties can be prepared [87].

Cotton, polyester, and polyamide textiles were coated with silica nanoparticles containing layers of three triarylmethane dyes (the cationic *Malachite Green*, the anionic *Guinea Green*, and the non-polar *Reflex Blue 61*) which were incorporated on the surface by the sol–gel method. The dyes were added to an acidic solution of tetraethoxysilane (TEOS) in ethanol and some water. The stability of the dyes was enhanced by incorporation into the silica layer [88].

Nanosols affected the mechanical and thermal stability and the repellent and optical properties of textiles, providing antistatic and bioactive coatings with a high potential for many new applications, e.g., in medicine [89].

Spherical silica powders with uniform, submicron grain diameter, prepared by the sol–gel method, were coated with nanoparticles (NPs) of metallic silver. Silver-doped silica powders (SiO_2_/Ag^0^) exhibited antimicrobial properties and can be used for the fabrication of thin-film coatings on textiles, e.g., with bacteriostatic properties [90,91]. Alternatively, by solvothermal methods (carried out in an autoclave with thermal or microwave heating) Ag and Ag/SiO_2_ sols were prepared, containing silver nanoparticles (NPs). A reduction of silver salt combined with the hydrolysis of (alkoxy)silanes during the solvothermal process gave Ag/SiO_2_ nanocomposite sols. The reduction of the silver salts was performed in ethanol in the presence of poly(vinylpyrrolidone) (PVP) at temperatures >120 °C. Ag NPs provided antimicrobial properties of modified viscose fabrics [92]. Thin layers of yellow/brownish silver NPs on polyamide (PA) surfaces were also obtained by a reaction of Tollens’ reagent with glutaraldehyde. Thus, PA textiles coated with Ag NPs exhibited excellent antimicrobial activity, great durability, and unchanged efficiency even after 30 laundry cycles [93].

The silica nanosols, modified with the addition of 3-glycidyloxypropyl(triethoxy)-silane (GPTES), silver nitrate, copper acetate, and hexadecyltrimethylammonium-*p*-toluolsulfonate (HTAT), were also used for the preparation of textile materials having antimicrobial properties. The modification process was carried out by the dip-coating of textiles (in a solvent containing 90% water), followed by thermal treatment at 80–180 °C. A decreased growth of bacteria (*Bacillus subtilis and Pseudomonas putida*) and fungi (*Aspergillus niger*) with increasing amounts of the antimicrobial agent supported in the coating was observed. The incorporation of GPTES enhanced the stability of the coating solutions and the biocidal effect of the coatings. In the case of a combination of the biocides (Ag, Cu, or HTAT) against both bacteria and fungi, a synergistic effect was observed [94].

Long-term stable sols of ZnO and composite Ag/ZnO NPs were prepared by the reduction of silver salt on the surface of ZnO NPs, dispersed in isopropanol. The prepared coating agent was combined with inorganic–organic hybrid binder sols prepared from 3-glycidyloxypropyl(trimethoxy)silane (GPTMS) and (tetraethoxy)silane (TEOS) and used for textile treatment. Cotton and cotton/polyester fabrics resistant to washing were obtained by the ”pad–dry–cure” method. They exhibited very good antimicrobial properties against the Gram-negative bacteria *Escherichia coli* and Gram-positive *Micrococcus luteus* [95].

Cotton and cotton/polyester (50%/50%) fabrics were modified by the *pad–dry–cure* technique with hybrid coatings, based on ZnO, Ag/ZnO/chitosan (CS), TEOS, and 3-glycidyloxypropyl(trimethoxy)silane (GPTMS), which were prepared by the sol–gel method. All these nanocomposite materials (Ag/CS, ZnO/CS, and Ag/ZnO/CS) exhibited good antimicrobial properties against the Gram-negative *E. coli* and the Gram-positive *S. aureus* bacteria and *Micrococcus luteus*. They can be recommended for medical applications [96].

Micro- and nano-cellulose were chemically modified with various silanes through free hydroxyl groups and formed covalent bonds. The low number of free hydroxyl groups present on the cellulose surfaces reacted with silanes, thus promoting surface modification. An increasing number of research works concerning the silane modification of cellulose have been published in the literature [97].

The surface of the cotton fiber was modified by products of condensation reactions of 3-glycidoxypropyl(triethoxy)silane (GPTES), carried out in an ethanol–water medium. The Fourier-transform infrared (FTIR) spectra showed two additional peaks at 860 cm^−1^ (Si–OH symmetric stretch) and 1207 cm^−1^ (corresponding to Si–O–C bending), respectively, as a result of the reaction of the hydroxyl groups on the fiber surface with the EtO–Si groups of the silane. The tensile strength (TS) and softness properties of cotton were increased as a result of modification with silane coupling agents (SCA), owing to the formation of more flexible Si–O bonds between the cotton fiber and the SCAs. The absorption of moisture by the modified cotton fiber was lower than for the raw cotton fiber, and the swelling of the modified cotton fiber was decreased in the polar solvents but increased in the nonpolar solvents. The absorption of *reactive brown 10* and *reactive orange 14* by the modified fiber was higher than for the unmodified cotton fiber [98].

A jute/cotton blended yarn (flat knitted as a single jersey fabric) was treated with a mixture of enzyme and amino functional PDMS silicone finishing agent to increase the softness of the fabric. The combined enzyme and amino silicone finishing treatment had significantly changed the one-way moisture transport capacity (OWTC) of the finished knitted fabric, which was rated as “very good” as compared to “poor” (for raw jute yarn), while the overall moisture management capacity (OMMC) rating was also increased from “very poor” to “good” after finishing. The finishing process changed the fabric properties from a water-repellent fabric to a water-absorption fabric. Thus, the properties of the jute fiber were modified through the silicone finishing treatment in order to increase its utilization as a component of day-to-day textile goods [99].

One step, eco-friendly treatments in textile finishing processes by nanotechnological sol–gel methods, in the same bath at one step using a low concentration of precursors, provided multifunctional properties of textile materials: flame retardancy, water and oil repellency, ultraviolet (UV) protection, self-cleaning, washing durability, textile comfort, antibacterial, antiwrinkle, and good physical properties. The sol–gel technology has many advantages in comparison with conventional textile finishing processes and is based on the application of metal salts or inorganic metal alkoxides to organic textile materials. Moreover, the sol–gel technology is an economical, ecological, and environmentally friendly one-step process, using a low concentration of nonhalogenated chemicals without formaldehyde release in comparison to conventional processes. The sol–gel technology is expected to be frequently used in textile finishing processes and promising studies for further developments in the future [85].

Nanocoating finishes by the sol–gel methods allowed to form thin (100–300 nm) and elastic coatings, chemically or adhesively bonded with the fiber’s surface, which provided modified textiles with improved properties and multifunctional characteristics, including the textiles’ performance durability, antiwrinkle, anticrease, barrier properties against UV radiation, bioactivity, antimosquito protection, and durable press or easy-care effect. The recent progress of sol–gel technology in textile finishing includes oil/water separation, photocatalytic self-cleaning capabilities, self-sterilizing, and heat storage, as well as photochromic and thermochromic color changes [85,86,98].

The hybrid SiO_2_/Al_2_O_3_ sol, synthesized from two precursors, 3-glycidoxypropyl-(trimethoxy)silane (GPTMS) and aluminum isopropoxide Al(Oi-Pr)_3_, was utilized for the thin-coat finishing of polyester/cotton woven fabrics of fiber blends (PET/cotton, 67:33). The fabric abrasion resistance was significantly increased, and the susceptibility to form pilling was almost completely eliminated. Moreover, the fabric was resistant to prolonged washing [100,101,102].

The surface of silica microspheres, which were prepared by the sol–gel method, was modified with antibacterial metallic silver and fungicidal copper nanoparticles (NPs). The obtained bioactive SiO_2_/Ag + Cu NPs (with anti-microbial properties) were incorporated into thin polymeric coatings, composed of hydrophilic polyurethane with a complex structure, which was prepared in situ on the surface of the textile PET fabrics. The basic functional properties of the modified coating materials (high resistance to water penetration, wind-proofness, and good hygienic characteristics, determined by means of water vapor permeability) have not deteriorated. Monodisperse silica microspheres were prepared according to the Stöber procedure [103] from a solution of tetra(ethoxy)-silane (TEOS) in ethanol, which was hydrolyzed in the presence of ammonia as a catalyst. The evaporation of water and alcohol gave dry, white powdered homogeneous submicrospheres. They were subsequently doped with metal ions from aqueous solutions of silver nitrate (AgNO_3_) and copper nitrate [Cu(NO_3_)_2_·2.5H_2_O] which were added to the submicrosphere suspension in appropriate weight ratios, followed by stirring and drying. After the thermal reduction, the silica still remained in an amorphous form [104].

The doping of the hybrid Al_2_O_3_/SiO_2_ sol with functional nanoparticles, Ag, Cu, and TiO_2_ (0.2 and 1 wt.%), did not deteriorate the strength properties of the prepared xerogel coatings. PET/cotton (67/33) fabrics coated with the above antimicrobial agents showed excellent bioactivity: an 83–92% reduction in the bacteria (*Escherichia coli* and *Staphylococcus aureus*) and an 87–93% reduction in fungi growth (*Candida albicans* and *Aspergillus niger*), as well photocatalytic self-cleaning and barrier properties against UV radiation (UPF >> 50) (five-functional finish) were achieved [103,104,105]. Multifunctional coatings on cotton fabrics using TiO_2_ and TiO_2_/SiO_2_ NPs, derived from TEOS and GPTMS, gave fire resistance, UV protection, and water-repellent properties [106].

The mixture of (tetraethoxy)silane (TEOS) and 2,4,4′-trichloro-2′-hydroxydiphenyl ether (triclosan, TC) or with quaternary (hexadecyl)trimethyl ammonium bromide was used for the modification of a cotton fabric, providing hydrophobic and antifungal properties against five mold species which cause the degradation of cellulose: *Chaetomium* sp., *Aureobasidium* sp., *Paecilomyces* sp., *Aspergillus* sp., and *Penicillium* sp. A silica layer formed from TEOS on the surface of the textiles increased hydrophobicity. The fabrics modified with TEOS and TC showed a higher antifungal resistance. The multifunctionally modified textile materials may find medical applications, e.g., for wound dressings, protective clothing, insoles, or as filters [107].

Different textile fibers (viscose, polyester, polypropylene, polyacrylonitrile, and wool) were also treated with solutions of (ethoxy)tetrasiloxane oligomers (”ethyl silicate 40”) at room temperature. The samples of polyester non-woven fibers modified with 2.5% oligoethoxy(4-benzylcarboxyphenyloxy)siloxane showed antimicrobial activity, i.e., intrinsic bacteriostatic properties [108].

Solutions of the precursor siloxane *N*-halamine monomers or polymers or *N*-halamine hydantoinyl epoxide derivatives were used for the preparation of the biocidal siloxane and epoxide coatings on cotton and polyester fabric (PET) surfaces by the soaking method [109].

The effect of the structure of silicone grafted with polyethers [containing poly(ethylene glycol) (PEG) or polypropylene glycol (PPG) side groups] on selected functional properties of cotton fabric rinsed in conditioners containing the additives was studied. Formulations of fabric softener containing two compounds with the comb structure (PEG/PPG-14/0 *Dimethicone* (i.e., dimethylcyclosiloxane oligomers or polydimethylsiloxane) and PEG/PPG-20/20 *Dimethicone*) and one agent of the block structure (Bis-PEG/PPG-20/20 *Dimethicone*) were used. The soft hand effect was observed after cotton rinsing in fabric softeners containing the block copolymers Bis-PEG/PPG-20/20 *Dimethicone*, while the highest fabric re-wettability was observed for the conditioner enriched with a compound of the comb structure (PEG/PPG-20/20 *Dimethicone*) [110].

Cotton fabrics treated with polyether and amino functional silicone softener (ETSO-PEA) had better whiteness than samples treated with commercial telechelic amino-propyl-terminated polysiloxane (ATSO). According to SEM images the ETSO-PEA- and ATSO-treated cotton fabrics, they showed significantly improved smoothness of the surface compared with the untreated samples. The incorporation of the polyether groups in ETSO-PEA increased the wettability and hydrophilicity of the fabrics in comparison to the ATSO-modified samples. These differences between the ETSO-PEA- and ATSO-treated cotton fabrics were explained as the effect of the molecular structure of silicones and their arrangement on the surface of the fabrics. The incorporation of polyether groups enhanced the wettability and hydrophilicity of the fabrics but slightly reduced the softness. The application of functional silicone softener distinctly improved the comprehensive performance properties of the fabrics: good softness, smoothness, hydrophilicity, and wettability [111].

The modification of polyester (PET) fibers with the silicone-acrylate adhesive increased the percentages of dye fixation and the color strength. The excellent color fastness (≥level 4) in the low-emissivity printing process was achieved. A residual dye concentration was decreased by ~19 times as compared with traditional direct printing, while the effluent wastewater drainage was lowered by 76.9% [112].

A fabric treated with Cu-doped TiO_2_, with the addition of oligosiloxanes prepared by the hydrolysis of TEOS, showed photocatalytic activity and excellent self–cleaning properties [113]. Additionally, CuO-doped TiO_2_ exhibited higher photocatalytic activity than TiO_2_. The fabrics modified with CuO/TiO_2_ composites, encapsulated clove oil, and coated on fabric exhibited better self-cleaning properties than blank fabric and also an anti-mosquito function [114]. Similarly, TiO_2_/SiO_2_/graphene oxide nanocomposite coatings were prepared on a polymeric in one step. The samples also saved their good photocatalytic properties even after 10 washes. The obtained NCs showed significantly better visible light self-cleaning performance than NCs containing only TiO_2_ [115].

Chemical modifications of cotton-based composite coatings were carried out by radiation-induced graft polymerization of 3-methacryloxypropyl(trimethoxy)silane in order to improve the adhesion of the TiO_2_ NPs to cotton. Through radiation-induced graft polymerization and sol–gel technology was prepared a new type of multifunctional cotton fabric, coated with a poly-siloxane–TiO_2_ hybrid, which exhibited UV resistance, switchable photo-induced water–oil separation, superhydrophobicity–superhydrophilicity, and self-cleaning properties [116]. Alternatively, in order to create durable functional properties, TiO_2_ NPs were functionalized with 3-glycidoxypropyl-(trimethoxy)silane and 3-(methoxysilyl)propyl(dimethyl)octadecyl ammonium chloride [117].

For the chemical and physical modification of textile fibers with TiO_2_ nanoparticles and nano-/micro-structures, the applications and performance of TiO_2_-modified textile materials have been described in an excellent and comprehensive review [118]. Different functional silanes and polysiloxanes were very often applied in these modifications of the properties of TiO_2_, which is one of the most attractive nanomaterials for the functionalization of textile materials due to its unique physicochemical, electrical, and optical properties, photocatalytic self-cleaning, antimicrobial activity, UV protection, hydrophobicity, thermal stability, nontoxicity, low cost, flame retardancy, and electrical conductivity of modified textiles. The photocatalytic activity of TiO_2_ in visible light was enhanced as a result of its surface modification, e.g., multiphase ion doping, metal doping/loading, heterojunctions, coupling with other semiconductors, and surface sensitization [118].

Silver nanowires (AgNWs) and two silanes: 3-aminopropyl(triethoxy)silane (APTES) and (diethoxy)dimethylsilane (DEDMS) were used for the functionalization of meta- and para-aramid fabrics, which were first pre-treated in a low-pressure air radio frequency (RF) plasma, followed by coating with polydopamine. The modified fabrics showed good hydrophobic properties (contact angle of 112–125°) [119].

The highly homogeneous borosiloxane hybrid silica sol, prepared from (tetraethoxy)silane (TEOS) and boric acid B(OH)_3_, was used for the improvement of the flame retardancy (FR) of silk fabric. The limiting oxygen index (LOI) values for the treated samples increased from 25.0% to 32.0% [120]. The FR properties of polyacrylonitrile (PAN) fabric treated by a one-step sol–gel process with TEOS and polyphosphoric acid were also studied; the LOI value increased to 30.1 and the char residue was 69.5% [121].

A novel, comb-like polysiloxane copolymer (GPPDMS), containing phosphoric acid and guanidyl- groups, was prepared by grafting P_2_O_5_ and dicyandiamide from siloxane oligomers, which were formed by the hydrolytic polycondensation of 3-aminopropyl-(triethoxy)silane H_2_N(CH_2_)_3_Si(OC_2_H_5_)_3_. Cotton fabrics modified with 18.6% GPPDMS reached a value of LOI 31.9 and the highest reduction in the heat of combustion. EDS analysis indicated that a substantial amount of Si and P was incorporated onto the surface of cotton fabric after combustion and showed a synergic effect, improving its FR. The addition of GPPDMS decreased the degradation of the fabrics and the liberation of volatile compounds but affected the formation of the char, and also caused the good antimicrobial activity of the modified cotton fabrics against *Staphylococcus aureus* and *Escherichia coli* (96% and 97%, respectively) [122,123].

The incorporation of Si, P, and N onto the surface of cotton fabrics, as a result of modification with 3-aminopropyl(triethoxy)silane, 3-glycidyloxypropyl(trimethoxy)-silane, and guanidine phosphate by the sol–gel method caused a synergistic effect on the FR properties. Moreover, the values of the LOI determined for the modified fabric samples reached 45.7% [124].

A composite polypropylene/silsesquioxane (PP/POSS) nonwoven fabric with permanent electret was prepared through the melt-blown technique with corona charging. POSS accelerated the crystallization process during the nonisothermal cooling stage, acting as a nucleating agent. In comparison with PP nonwoven fabric, increased values of tensile strength (TS) and elongation at the break of the PP/POSS melt-blown nonwoven fabrics were observed. The morphology and the distribution of octavinyl POSS nanoparticles on the surface of the composite fibers were analyzed by a field-emission scanning electron microscope (FESEM) and wide-angle X-ray diffraction (WAXRD), respectively [125].

Oligo(dimethylsiloxanediol) (Polastosil^®^ M-200) and hydroxy ether polysiloxane graft copolymer: poly[dimethylsiloxane-*co*-[3-[2-[(2-hydroxyethoxy)propyl]methyl-siloxane, were used as plasticizers of PP and PLA nonwovens with excellent antimicrobial properties (due to the presence of copper silicate hydrate), which were fabricated by the melt-blown method [126,127].

A surface of polyurethane (PU) fabrics was coated with hydrophobic hybrid silica films, which were prepared from TEOS and 3-glycidyloxypropyl(trimethoxy)silane (GLYMO) by the sol–gel method combined with dip coating. The thermal stability was improved, and the textile feel of the PU fabrics was saved by GLYMO-based treatments. Scanning electron microscopy (SEM) images revealed that the PU surface and pores were completely modified with thin hybrid silica films, which were preserved for most of the samples [128].

The anionic waterborne polyurethanes (WPUs) were prepared from poly(tetra-methylene oxide) and hydroxyl-terminated PDMS as soft segments (SS), isophorone diisocyanate (IPDI) as hard segments (HS), and 2,2-bis-(hydroxymethyl)propionic acid as an ionic center. The tensile strength of the WPU-PDMS films decreased with the increasing content of PDMSs, and the water repellency reached 80%, which was equal to the capability of silicone rubber (SR). The obtained waterborne siloxane-containing PUs can be useful for textile and plastic coatings, sealants, leather finishing, and glass-fiber sizing [129].

Emulsions of new silicone-urethane copolymers, prepared by a reacting equimolar ratio of 2,4-toluene diisocyanate (TDI), polyethylene glycol (PEG) of a different molecular weight (300–6000 Da), and carbinol polydimethylsiloxane (CPDMS), at 100 °C for 90 min, were used as textile softeners. They caused an enhancement in the functional properties of treated cotton fabric tear strength, softness, anti-crease, and flexibility, and a reduction in the wettability and whiteness index. A cotton fabric modified with the emulsion formulation of CPDMS/TDI/PEG1000 softener finishing, containing triclosan, exhibited durable antibacterial properties [130].

After treatment with a reactive urethane–silicone agent (which was crosslinked with a blocked isocyanate and reacted with cellulose surfaces at 150 °C within 30 min.), the durability to washing, dimensional stability, and soft touch of cotton knit fabrics, were improved. The treatment of cotton knit fabrics with the urethane–silicone softener provided also excellent elasticity, flexibility and shear recovery, recovery against bending deformation, and soft and smooth surface characteristics, with a small coefficient of friction even after washing 20 times [131].

### Modification of Textiles Surfaces with Quaternary Ammonium (Alkoxy)Silanes

Silane and siloxane coupling agents (SCA) can react with many polymers and silanization with (alkoxy)silanes containing halogenalkyl or onium groups is often used for the surface modification of a wide range of polymeric materials, including textiles. During the hydrolytic polycondensation of (alkoxy)silanes, polysiloxane networks are formed, and surface coating can be achieved by the adsorption or covalent binding between polysiloxane and the surface of polymeric fibers. This way, antimicrobial coatings were often prepared on the surfaces of textile materials. The silanization of natural and synthetic fabrics with (alkoxy)silanes containing onium groups was applied a long time ago for the fabrication of commercial polymer-based materials having antibacterial properties [132,133,134,135,136].

Textiles treated with cationic 3-(trimethoxysilyl)propyldimethyloctadecyl ammonium chloride (**1**) [132,137,138,139] were known under the trademark *BioGuard* in the late 1970s in the last century. They had excellent antimicrobial activity against many Gram-positive, Gram-negative, or odorous microorganisms [140]. Many kinds of textile materials can be modified during antimicrobial treatment: cotton/polyester blends, nylon, outerwear fabrics, underwear, mattress ticking, carpeting, throw rugs, etc. Cationic (trimethoxy)silane (**1**) was applied for the modification of PET fabrics [134] and non-woven Sontara fabrics [136]. PET non-woven fabrics coated with a polysiloxane network [formed by the hydrolytic polycondensation of 3-(trimethoxysilyl)propyldimethyloctadecyl ammonium chloride **(1)**] showed very high efficiency against both Gram-positive *S. Aureus* and Gram-negative *K. Pneumonia* (more than 99.9%), and also against Gram-negative *E. coli* (>99.5% killing rate).

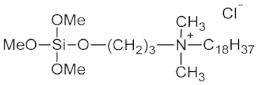
(**1**)

Onium salts were widely used, not only as biocidal agents but also for the antimicrobial treatment of various surfaces, for example, with cationic (trimethoxy)silane (**1**), containing quaternary ammonium groups (QAGs) [136,137,138,139]. Coatings of polysiloxane networks, derived from (**1**), interacted with hydroxyl groups on the surfaces of textile materials containing QAGs and exhibited antimicrobial activity against a broad range of microorganisms including bacteria (*Escherichia coli*, *Streptococcus faecalis*) [136,137,138], fungi (*Aspergillus niger*, *Aspergillus flavus*, *Aspergillus versicolor*, *Penicillium funiculosum*, *Chaetomium globosum*), and algae [137]. Similarly, zol obtained by the hydrolytic polycondensation of (**1**) was used for the treatment of polyurethane (PU) foams [135]. The PU foams coated with biocidal polysiloxane films exhibited an efficiency higher than 99% against many Gram-positive bacteria. Moreover, the antimicrobial activity of both fabrics and PU foams remained stable after repeated washing with various detergents [135,140].

Cotton textile materials were also modified by the covalent binding of (alkoxy)silane containing phosphonium groups with different alkyl substituents [R = −C_2_H_5_, −C_4_H_9_, −C_6_H_13_, −C_8_H_17_) of the structure (**2**)] via reactive hydroxyl groups of cellulose [141] and cellulose filters [142]. They showed higher antibacterial activity against *S. aureus* than against *E. coli*. The highest biocidal activity was reached with the use of (alkoxy)silane with the longest n-octyl chains [142,143].

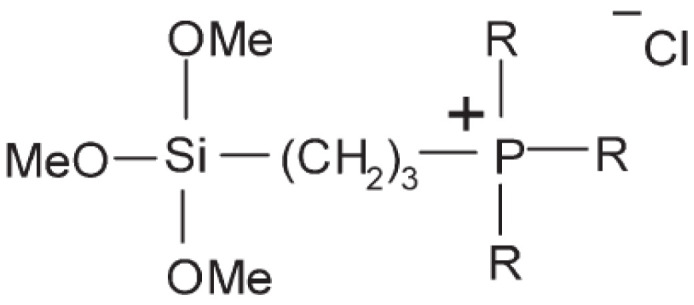
(**2**)

R = −C_2_H_5_ (**2a**), −C_4_H_9_, (**2b**), −C_6_H_13_, (**2c**), −C_8_H_17_ (**2d**)

Silyl quaternary compounds have been used for treatments of synthetic fibers for a relatively long time. Depending on the kind of fibers, quaternary ammonium siloxanes (QAS) bind with modified surfaces by adhesive or chemical means [144].

The modification of surface properties with organofunctional silanes (QAS) involves two processes:

(1) a very rapid substitution of protons from water on the surface by the cation of QASs, in an ion-exchange process;
(2) a hydrolytic polycondensation of alkoxy silane groups of QASs leading to the covalent bonding to the substrate surface [145].

The modification of textiles with commercial 3-(trimethoxysilyl)propyldimethyl-octadecyl ammonium chloride:

(MeO)_3_Si[–CH_2_CH_2_CH_2_N^+^(Me_2_)C_18_H_37_]Cl^−^ (QA-TMS)

led to antimicrobial properties. Coatings based on silicones containing Si–O–Si units had low sliding angles below 10° [146], low surface tension and glass transition temperature [133,147]. PET fabrics were coated with (**QA-TMS**) by a simple pad–cure technique, followed by hydrolytic polycondensation in acidic medium at temperatures exceeding 100 °C. The obtained coatings on the PET surfaces were relatively hydrophobic and exhibited rewetting times at higher than 100 min. The hydrophilic coating with a rewetting time of 2.8 min was formed when **QA-TMS** was initially hydrolyzed under alkaline conditions, followed by polycondensation under acidic conditions. Superior anti-microbial properties were obtained for all coatings based on **QA-TMS** on PET substrates. Next, coated fabrics were dyed with direct dye [134]. Quaternary (3-(trimethoxysilyl)propyl(dimethyl)octadecyl ammonium chloride) was also used for the antimicrobial functionalization of cotton/elastane fabrics, which were applied for clothing [148]. The coating of cotton textile with quaternary ammonium-modified (triethoxy)silane by the sol–gel process gave a transparent film with significant anti-microbial properties against *S. aureus* and *E. coli*, even after 15 washing cycles and a permanent non-leaching antibacterial finish effect [149].

Before treatment with siloxanes containing onium groups, the relatively inert surfaces were pre-activated. For example, the activation of silicone rubbers by gas plasma allowed the incorporation of hydroxyl groups onto the surface layers of widely used implantable biomaterials [150,151,152]. Silicone rubbers, activated by plasma on the surface, were treated with (trimethoxy)silane (**1**), resulting in materials that showed high antimicrobial activity against both Gram-negative and Gram-positive bacteria in in vivo experiments [150,151,152].

## 4. Applications of Silicone Elastomers and Rubbers for Fabrication of Thin Coatings on Textile Materials

Silicone elastomers (SEs) are available as room temperature vulcanizing (RTV), heat temperature vulcanizing (HTV), or liquid silicone rubber (LSR) systems. Liquid RTV silicone compositions crosslink with the formation of gel products intended for adhesion, sealing, coating, encapsulation, or potting applications. They are offered as one- and two-component products, which undergo crosslinking in the presence of atmospheric moisture or during heating, via condensation or addition (hydrosilylation) reactions. LSR silicone compositions usually have a relatively low viscosity range and are additionally crosslinking materials, most often designed for short-cycle time injection molding and automatic processing. For coating applications, HCR products are diluted with solvents. Another kind is High Consistency Rubber (HCR), which was designed for typical processes: extrusion, calendering, and molding (including compression, transfer, and injection). Some silicone elastomeric products are supplied as masterbatch compositions containing the peroxide initiator or a hydrosilylation catalyst in one or two ingredients. At present, additional crosslinking is the preferred technology for the coating of textile materials with silicones. One-part addition crosslinking mixtures are also available. The hydrosilylation reaction of RTV and LSR products is very fast, and its work life is adjusted to the consumer’s needs. The vulcanization conditions are between less than a minute at room temperature (with approximately a 20 s pot life) and several minutes at 150 °C (with a pot life of days). Without solvents, no by-products are liberated during the vulcanization. The resin-type Silicone Liquid Elastomer (SLE) products are often used for textile coatings. However, LSR or RTV mixtures for textile coating are usually cured at elevated temperatures. They are a very good choice when a smooth and non-blocking surface is required [153].

The RTV SRs are used for the impregnation and coating of a variety of fabrics, papers, wires, and cables (e.g., for automotives). Papers and textile materials impregnated and coated with SRs have a lot of flexibility, moisture resistance, and good dielectric properties. The SRs, practically non-wettable with water, can be used for coating technical textiles and paper (e.g., for the manufacture of self-adhesive labels), and for sealing construction elements. Owing to their oil and heat resistance and long-term durability, they are often applied as automotive components. LSR parts show a low compression set and excellent steam resistance. The liquid SRs (LSR) are used for injection molding or for the coating of textile materials. They are applied especially in high-tech fields, e.g., as gaskets, automotive parts, and hardware in microwaves, and due to their conductivity and fatigue resistance, they are ideal materials for electronic interfaces on keyboards or touch pads [154,155].

For many years, silicones have been used for the fabrication of a continuously growing variety of industrial textiles, mainly due to specific performance requirements. Silicone rubber (SR) coatings are flexible at low temperatures (−85 °C) and show superior heat and cold resistance with good weathering performance, UV light stability, water repellency, good electrical properties, and fire retardancy. Solventless processing is most often performed, and silicone systems can be processed with knife coating, dip coating, extrusion coating, calendering, and spray coating equipment. Different types of silicone rubbers were developed for a large range of fabrics, and owing to their unique physical properties, they find numerous interesting applications in industrial textiles, e.g., as architectural fabrics, parachutes, panel heaters, and nonslip coatings [15].

Silicone rubber is a high temperature-resistant and an important insulation coating material. SR has excellent weather resistance and it is also used as balloon coating for cars’ buffer airbags, effectively resistant to aging. Silicone rubbers seem to be the ideal choice for the balloon coating of aerial vehicles. A composite composed of fiberglass coated with the SR on the surface, and then through the vacuum spray technology to foil an aluminum layer, was used as a silicone rubber aluminum thermal insulation cloth for −60 to ~250 °C long-term working, generally used in aircraft cockpits, umbrellas, booster cabins, and other insulated parts. Silicone-coated glass fiber fabrics are used as high-temperature anticorrosion conveyor belts, some high-temperature-protective gloves, asbestos insulation blankets, high-temperature service fabrics, glass fiber fire cloth, and other goods. Such composites can be applied as fire-resistant insulation materials [156].

The textile fabrics coated with silicone rubber have high electrical insulation properties (i.e., resistance to high voltage performance), combined with good adhesive and anticorrosion properties, which are very useful in insulating materials, sleeving, and other products. Screen printing on textiles using silicone rubbers is also widely used, leading to brightness, waterproofing, high peel strength, abrasion resistance, anti-skid, and high-temperature resistance. Since silicone rubbers are easily colored, they are widely applied in the manufacture of clothing accessories, shoes and hats, luggage and other handbags, and other textile goods. It also has a good vibration absorption capacity. The application of silicone rubber provides anti-skid properties to clothing, e.g., women’s underwear, laces, stockings, etc., preventing clothing slippage. Silicone rubber coatings can ensure a rounded, right-angle effect, three-dimensional sense of strength, excellent tear resistance, and comfortable contact with the human body, both to provide the beauty of clothing and its functional role [156].

The glass fiber fabric-silicone-laminated cloth is a high temperature-resistant, high-strength, high-performance, and multi-purpose textile composite material, fabricated by special technology. It can be widely applied in chemicals, petroleum, equipment, machinery, metallurgy, electrical insulation, construction, aerospace, and other fields. Silicone cloth can also be used as a fire-resistant insulation material. Except for high-temperature insulation, its main performance characteristics include high strength, softness and toughness, chemical corrosion resistance, oil and water resistance, and tailor ability. The silicone cloth was also used for thermal gaskets. It can improve thermal conductivity, coupled with the strength of the silicone rubber itself. Silicone glass fiber cloth coated with nickel-chromium can be utilized in aviation equipment and instrumentation, often used in heating elements and electric blankets. Its role is to control the operating temperature of the instrument.

The silicone rubber surface has high friction, and it is often coated on surfaces of some anti-skid pads. An anti-skid effect is better for bath mats, car mats, etc. In some special clothing, the body needs to focus on the protection of parts coated with a certain thickness. A certain shape of silicone rubber can improve people’s wear safety, such as racing suit knees, elbows, shoulders, and other parts. The silicone rubbers are nontoxic, they do not cause pollution, and they are also suitable for the replacement of the other additives in the finishing process, leading to the improvement of products’ quality [156].

The current technology enables dyeing the silicone rubber, and it can change the color of silicone-coated fabric to change the clothing color. A silicone surface is not easy to contaminate. It is suitable for winter garments fabric [156]. Usually, 0.5% to 2.0% of color pastes is used for pigmenting the additional cure of LSR, depending on the desired depth of color [153].

Natural and synthetic textiles, especially highly elastic clothes, were coated with a two-component liquid silicone rubber (SILASTIC™ LCF 5120 Liquid Silicone Rubber) by a textile screen printing method, providing many benefits [157]:

flowable, fast curing,
glossy, medium hardness, high elongation,
excellent unprimed adhesion to polyamide fabric,
soft, flexible, high-strength coating.

Moreover, textile materials were easily pigmented and ironable and did not contain PVC, phthalates, organic solvents, or formaldehyde.

Four kinds of composite polymeric membranes: PDMS/poly(vinylidene fluoride (PVDF), poly(phenyl methyl siloxane) (PPMS), poly(ethoxy)methylsiloxane (PEOMS), and poly(trifluropropyl-*co*-methylsiloxane) (PTFMS) were formed on a PVDF matrix which was reinforced with a nonwoven polymeric fiber. They were used for the separation of ethanol from ethanol/water mixtures. The hydrophobicity of the membrane’s surface was evaluated by contact angle measurements. The separation properties of these membranes were strongly dependent on the silicone rubber composition [158].

Three kinds of silicone-based tubular materials: silicone foam rods, silicone rods, and silicone hollow tubes were applied for the reinforcement of polyester spacer fabrics (commonly used as cushioning materials) by a knitting method. The compression properties of the spacer fabrics at an initial compressive strain of 10% were independent of the presence of the inlaid tubes. Young’s modulus of the inlaid tubes was proportional to the value of the compression. The spacer fabric inlaid with highly elastic silicone foam tubes absorbed more compression energy, while that inlaid with silicone tubes of higher tensile strength had higher compressive stiffness [159].

The simultaneous crosslinking and grafting of the emulsions of the silicone elastomers based on poly(dimethylsiloxane-α,ω-diol) (containing 0.02–1.7% of reactive terminal silanol groups) with the emulsion of poly(methylhydrosiloxane) oil (PMHS) on a knitted fabric were carried out in the presence of aqueous solutions of zinc and/or tin salts, containing tertiary or quaternary aminoalcohols as catalysts during the drying and heating process [160].

The SR-titania composite fibers were formed from hydroxyl-terminated PDMS (M_n_ 46,000 g/mol), tetraethoxysilane (TEOS), and titanium isopropoxide Ti(OiPr)_4_, by combined sol–gel and electrospinning methods, followed by heating at 250 °C for 3 h. The tensile strength (TS) and modulus of the TiO_2_/PDMS fibers were proportionally dependent on the PDMS content (10–30 wt%), while their photocatalytic properties were strongly increased with the increasing TiO_2_ content [161].

### Textiles for Silicone Rubber Composites

On the other hand, since silicone elastomers and SRs exhibit low strength as compared to classical organic rubbers, and thus textiles are often used as reinforcement agents, except for powdered fillers. The radical curing of SRs with peroxides is one of the methods used for this purpose and is particularly used for aerospace applications but also for many different industries. Good adhesion between the fabric and SR was achieved, and vinyl methyl silicone rubber (VMQ) with excellent thermal stability is widely used for high-temperature applications and heat protection. Methacryloxy(trimethoxy)silane was very often used, especially for the modification of the surface properties of aramid, nylon, polyester, and glass woven fibers, and also polyurethane, acrylics, many thermoplastics (PVC, polyolefins), and some inorganic fillers (e.g., silica and vermiculite) [162].

## 5. Superhydrophobic Thin Coatings on Textile Materials Obtained by Silanes, Siloxanes, or Silicone Treatment

The surface properties of textile materials can be modified, leading to decreases in their surface tension and hydrophobicity against liquids [163].

**Figure 5:**
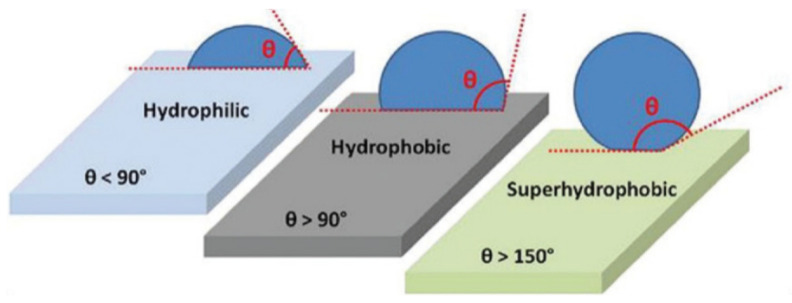
A graphical illustration of hydrophilicity–hydrophobicity properties of materials as ranges of contact angles [86,164]. Copyright MDPI, Basel, Switzerland—under the terms and conditions of the Creative Commons by Attribution (CC-BY) license; http://creativecommons.org/licenses/by/4.0/.

Multifunctional fabrics with super antiwetting (superhydrophobic) properties and controlled adhesion find many special industrial applications, e.g., for oil–water separation, self-cleaning, asymmetric/anisotropic wetting for microfluidic manipulation, air/liquid directional gating, and micro-templates for patterning. The hydrophilicity and hydrophobicity of substrate surfaces were characterized by the values of the contact angles (θ), which are illustrated in Figure 5 [164].

Various chemical additives were used for the finishing of the hydrophobic textiles [165,166,167,168]:

- insoluble metal and fatty acid salts synthesized in situ,
- waxes dissolved in organic solvents,
- quaternary pyridine derivatives,
- metal–organic complexes (e.g., chromium complex of fatty acid),
- reactive (methylol)urea or (methylol)melamine derivatives containing hydrophobic fatty acid substituents and silicones.

The sol–gel technology is very often applied for the hydrophobic and superhydrophobic treatment of the surfaces of many kinds of textile materials.

Water-repellent textiles were prepared by coating with different kinds of silica sols. The chemical compositions of pure silica sols and 3-glycidoxypropyl(triethoxy)silane-derived complex silica sols were modified with alkyl(trialkoxy)silanes, polysiloxane derivatives, and fluorine-containing silane. The polyamide fabrics and a polyester–cotton blend were used for the coating process with the above sols. Their hydrophobic properties were determined by contact angle measurements, which were in the range of 96–142°, and increased with the increasing concentration of the alkylsilane in the silica sol and the length of the alkyl chain. Similarly, textile coatings having high hydrophobicity were obtained by treatment with hydrophobic polysiloxane or fluorine-containing silanes. High wash-out stabilities were achieved only by using silica sols containing fluorine compounds and hexadecylsilane derivatives. Long-chain alkyl(trialkoxy)silanes can be used as substitutes for fluorine compounds for the hydro-phobization of textiles [169].

The textile comfort parameters, water uptake, air permeability, and stiffness, of viscose and polyamide, dip-coated with silica sol and a hybrid polymer-modified silica sol, were investigated by B. Mahltig et al. A sufficient low stiffness, an appropriate textile comfort, and the water uptake were observed only when low-concentration coating solutions were used. Hydrophobic textile properties were prepared after modification with perfluorooctyl(triethoxy)silane. Antimicrobial functionalization was carried out with a silver-containing sol. Concentrations ≤ 1% were necessary in order to obtain water repellency or antimicrobial activity [170].

Textile materials with superhydrophobic surfaces have also found biomedical applications owing to their ability to repel blood as well as reduce bacterial or viral adhesion to surfaces [171,172,173,174]. For instance, cotton fibers which were modified by Tomšič et al. with repellent coatings (prepared by the sol–gel technique) showed antibacterial properties [175].

Hybrid organic–inorganic sol–gel precursors containing longer aliphatic substituents: hexadecyl(trimethoxy)silane, octadecyl(trimethoxy)silane, and dimethyloctadecyl-[3-(trimethoxysilyl)propyl] ammonium chloride, and also octamethyltrisiloxane, poly(dimethylsiloxane) and polyvinylsilsesquioxane, stearic acid, poly(hexafluorobutyl-metacrylate), and other fluorocarbons, were very often applied for the preparation of the hydrophobic thin coatings on the textile fibers [117,118,176,177,178,179,180,181,182,183,184,185,186].

Hydrophobic finishes with silicones are very advantageous compared to the previous generation of hydrophobic compounds. Wash-resistant finishes have been obtained. The products exhibited a silky, “silicone” grip. They are used in the form of water micro- or nano-emulsions. The polysiloxane chain coated on the fiber is crosslinked with organometallic catalysts, e.g., zirconium compounds [166]. Depending on the kind of catalyst used, the following effects were achieved:

- a different degree of the crosslinking of the polysiloxane,
- varied waterproof effect,
- varied product grip.

The hydrophobic methyl groups (–CH_3_) of the silicone chain are located outside of a surface, and the silicon atoms are adjacent to the fiber surface. In order to obtain a good hydrophobic finish, all impurities (mainly surfactants) must be removed from the impregnated fabric. Silicone hydrophobic finishes are used for textile fabrication by padding, drying in air at 100–120 °C, and then heating at 150–155 °C for 3–4 min. Mainly aqueous emulsions containing poly(dimethylsiloxane) or its derivatives (as active ingredients) and emulsifiers, hydrotropes (e.g., glycols), and water are used. Currently, the polysiloxanes used in textiles for waterproof finishes are mainly Si–H functional linear copolymers (PMHS) composed of dimethylsiloxane and methylhydro-siloxane units, having a general structure of Me_3_SiO(Me_2_SiO)_m_(MeHSiO)_n_SiMe_3_ [16,187,188,189].

In alkaline or strongly acidic conditions, the ≡Si–H bond in PMHSs can be rapidly hydrolyzed and is therefore normally buffered at pH 3–4 and stabilized with certain organic compounds. The ≡Si–H bond undergoes hydrolysis with water to form a silanol group (≡Si–OH), which can then undergo a homocondensation reaction with another silanol group or a H-silyl group (≡Si–H) leading to crosslinked structures [164]. During the finishing of textiles, PMHSs containing Si–H groups bound to the Si atom of the polysiloxane chain require reheating at a temperature of 120–150 °C for up to several minutes. Using PDMSs requires heating at a temperature of about 100 °C higher and for a much longer time, but the grip of the finished fabric is soft and pleasant. The greater proportion of unsubstituted Si–H groups in the polysiloxane chain causes deterioration of the grip. The Si–H groups take part in the crosslinking of the polysiloxane chains, creating a developed, three-dimensional structure on the fiber surface. However, polysiloxanes containing longer alkyl and aromatic groups are used for many specialized applications. In the case of modified polysiloxanes with reactive groups, a durable finish of natural fiber products can be obtained. A batch finish is performed in a water bath containing 1–2% polysiloxane and a cationic agent to neutralize the negative zeta potential of the fiber. The optimal finish is usually obtained approximately 24 h after reheating. Good results were obtained by using polysiloxanes together with resins for finishes, reducing cellulose crumbling [166].

A great number of papers concern the preparation of hydrophobic and superhydrophobic surfaces on textile materials [190,191,192,193,194,195,196,197,198,199,200,201,202,203,204,205]. Some other publications are discussed in this article below. Silica-based superhydrophobic coating films, prepared at ambient temperature on cotton samples through the cohydrolytic polycondensation of the silanes mixture [*n*-hexadecyl(trimethoxy)silane (HDTMS), (tetraethoxy)silane (TEOS), and 3-glycidyloxypropyl(trimethoxy)silane (GPTMS)], were transparent and stable. The water contact angle (WCA) for the modified superhydrophilic cotton increased from 0° (before modification) to 141°. This nanocoating has found new applications for textiles and plastics. It is an environmentally friendly substitute of fluorine agents [206] (while some fluorochemicals and organic solvents have potential risks to the environment and human health) [207,208]. Similarly, superhydrophobic coatings on textiles were fabricated by dip-coating method with Stöber nanosilica, which was modified with *n*-hexadecyl(triethoxy)silane (HDTES). They showed very good water repellency, good chemical properties (e.g., towards acid and organic solvents and stability during laundering), environmental stability (against UV irradiation and external conditions), and excellent mechanical properties (especially abrasion and scratching resistance, an improved tensile strength, and elongation at breaking) [201].

The superhydrophobicity and abrasion stability of the modified textile materials depend on the kind of organosilane and its content in a bath, as well as the parameters of the dip coating process (e.g., concentration of nanocomposite, ultrasonication, and curing time). The superhydrophobic textiles exhibit superior mechanical, chemical, and environmental durability under optimal conditions, and also stable hydrophobic properties. The slightly reduced superhydrophobicity can be easily improved by dip-coating again in the same nanocomposite solution. They find many practical applications in different fields, e.g., for the separation of oils from water [209].

A highly durable superhydrophobic cotton fabric was also prepared by the hydrolytic copolycondensation of the ethanolic solution of TEOS and *n*-dodecyl(trimethoxy)-silane in the presence of ammonia (NH_3_∙H_2_O) accelerated with ultrasounds. The modified fabric coating showed both superhydrophobic and super-oleophilic properties simultaneously with a high water contact angle of 154 ± 0.5° and liquid repellency for common household liquids, outstanding stability in deionized water, various solvents, strong acidic/alkali solution, and boiling water, and good mechanical robustness after a sandpaper abrasion test. This fabric coating can be used in oil spill accidents for the oil/water mixtures (e.g., *n*-hexane, *n*-heptane, dodecane, and kerosene), and the separation efficiency was more than 96%. The obtained fabric maintained good properties even after 50 separation cycles with above 97% of separation efficiency for the both dodecane/water and *n*-heptane/water mixture. [210].

Superhydrophobic cellulose-based materials are non-toxic, biodegradable, and renewable, and can substitute polymers and plastics with hydrophobic properties, which are used in many industries. Despite its hydrophilicity, cellulose has many advantages as a substrate for the production of superhydrophobic materials, which can be widely used in self-cleaning, self-healing, oil and water separation, electromagnetic interference shielding, etc. They find applications as green-based materials [211].

The silica nanosol prepared by the Stöber method by the hydrolysis of TEOS in aqueous ethanol in the presence of ammonia and modified with perfluorooctylated ammonium silane coupling agent (PFSC) was used for the preparation of the superhydrophobic coatings composed on the cotton fabric. After being immersed in the silica sol ion in the silica nanosol, the fabric was dip-coated twice. The dip-coating process was repeated twice, which dried the fabric, which was soaked in the methanol solution of PFSC, followed by curing at 160 °C. The water and oil contact angles for the modified fabric reached about 145° and 131°, respectively, confirming a high hydro-phobicity and oleophobicity of the cotton fabric [212]. A complex coating of silica nanoparticles functionalized with amino groups onto cotton textiles followed by hydrophobization with 1H,1H,2H,2H-perfluorodecyl(trichloro)silane, stearic acid, or their mixtures gave also superhydrophobic surfaces with dual-sized surface roughness. Their contact angles (142–168°) were determined [213]. A composite superhydrophobic coating was also treated with dispersions of silica nanoparticles modified with 1H,1H,2H,2H-perfluorooctyl(triethoxy)silane (PTES) in fluorosilicone resin and was cured at room temperature. The morphology and topography of the composite coating were dependent on the silica content [214].

The fluoro-containing silica sols, formed by the alkaline hydrolysis of TEOS and the copolymer of hexafluorobutyl methacrylate and 3-methacryloxypropyl(trimethoxy)-silane (PHFA-MPS) in ethanol solution were used to impart the cotton textiles with excellent superhydrophobicity via a facile dip-coating method. The modified cotton textiles exhibited excellent superhydrophobicity with a WCA of 153.4° and good stability [215].

The one-step chemical modification of the cotton fabrics in solutions of bifunctional polysiloxanes, which contained in their structure (trialkoxy)silyl or glycidyl functional groups (capable of bonding to cotton), and fluoroalkyl groups also led to the superhydrophobic surfaces. The superhydrophobic properties of cotton fabrics (measured as the water contact angles by a drop profile tensiometry were protected even after multiple washings. Any decrease in the mechanical properties and color changes as well as no stiffening of the modified fabrics were observed [202]

C.H. Xue et al. prepared colorful superhydrophobic poly(ethylene terephthalate) (PET) textile surfaces with self-cleaning properties by chemical etching with a sodium hydroxide solution and coating with PDMSs. The original textiles, first washed with deionized water, followed by soaking in NaOH solution for 10 min., were then coated doubled-side by a polyethylene film and heated at 120 °C. Next, the textiles were rinsed with neutral water (pH 7) and dried in an oven. The etching of the solid PET fibers’ surfaces caused a decrease in the contact area between the water droplets and the textile surface and an improvement of the fiber hydrophobicity, which allowed the water to easily roll off the textiles. The PDMS-modified textiles exhibited excellent durability against solutions with different pHs and remarkable resistance to abrasion, washing, and exposure to UV light. The superhydrophobic surfaces were dyed by the conven-tional method or by thermal transfer printing [216].

Ethyl cellulose (EC) was crosslinked with epichlorohydrin (ECH) and complexed with silanized carbon nanotubes (Si-CNTs), giving reinforced porous ethyl cellulose (SEC) sponges, which were next coated on the surface with nanosilica, and subsequently modified with hexadecyl(trimethoxy)silane (HDTMS). These sponges showed outstanding mechanical strength, superhydrophobic and superoleophilic properties (WCA: 158°, oil contact angle 0°, and sliding angle: 3°), and the capability to absorb oils and organic solvents. After 50 separation cycles, the absorption capacity was 64 times its own weight, which slightly decreased to 86.4% of its initial value. The SEC sponges were recommended as an ideal absorbent for the cleanup of oil spillage [217].

Another superhydrophobic cotton nonwoven fabric was obtained by the graft plasma polymerization of oligosiloxanes [(Me_2_SiO)_4_ or Me_3_SiOSiMe_3_ (HMDSO)] under air atmosphere and plasma treated. Their surface morphology and contact angle were dependent on different process conditions. The surface characteristics of the modified cotton nonwoven were analyzed by SEM, EDS, and FTIR spectroscopy. The WCA of the modified nonwoven fabric increased up to 155°. It had an excellent self-cleaning ability and was used for oil–water separation, with a separation efficiency higher than 97%, which was repeated for at least 10 times. Moreover, it showed excellent stability against strong alkaline and acidic media [218].

A combination of inductively coupled plasma (ICP) and micro-mold casting enabled the preparation of gecko-inspired PDMS microfiber surfaces. Their larger adhesive force was the result of the higher surface energy counterface. The smaller dimension and lower duty ratio of microfibers on the PDMS led to the increased water contact angle (WCA) and the decreased sliding angle (SA), in comparison with a smooth PDMS surface. The biggest WCA (155°) and SA (7°) were observed for one sample, indicating the superhydrophobic properties. All samples showed the best wet self-cleaning efficiency [219].

Newer superhydrophobic, UV-blocking cotton fabrics were developed by coating with mixtures of poly(vinylsilsesquioxane) (PVS) and nano-TiO_2_. After curing, the titania nanoparticles (NPs) were supported in the PVS film layer on the surface of cotton fibers by covalent Ti–O–Si bonds. The UPF value of the treated cotton fabrics modified with 23.4 wt.% of an ethanolic dispersion containing PVS and TiO_2_ (5 g each in 100 mL of ethanol) increased up to 121.5 with the increase in the TiO_2_ NPs content and their scattering and absorbing properties of UV radiation. The mechanical properties of the composite coatings on cotton fabrics were significantly increased. The new class of UV-blocking, superhydrophobic cotton fabrics may find many practical applications as advanced UV-blocking textiles, self-cleaning materials, and stretchable electronic devices [180].

In a two-step modification process, highly hydrophobic and fire-resistant cotton fabrics were prepared. The surface of the cotton fabrics was first modified by the sol–gel process with aminopropyl(triethoxy)silane (APTES), guanidine carbonate, and ammonium dihydrogen phosphate. Next, it was impregnated with fluoro-functional silane or polysiloxane. Although the WCA reached only 141–143°, a substantial reduction in the flammability of the modified cotton was achieved, with very high values of the limiting oxygen index (LOI): 46–71%. An improvement in the thermostability was also obtained [220].

A flexible polytetrafluoroethylene (PTFE) membrane reinforced with silica (SiO_2_) was used for the preparation of a superhydrophobic coating on aramid fabric by the two-step technique. It showed excellent chemical resistance and self-cleaning properties. Owing to the low surface energy and rough micro/nanostructures, the hydrophobicity was significantly increased up to the WCA of 154° and rolling angle of 4°. The PTFE/SiO_2_-coated fabric exhibited breathable properties and could withstand over 300 cycles of abrasion and strong alkaline or acid treatment for up to 100 h. The PTFE/SiO_2_-coated fabric was recommended for the manufacture of individual protective clothing for emergency rescue applications [221].

Melt-blown (MB) nonwovens modified on a surface with silica (prepared by the sol–gel method) and 0.6 wt% of Si_3_N_4_ showed superhydrophobic (WCA 161.7°) and good bacteriostatic properties against *E. coli* and *S. aureus*, which increased >96% [222].

A laser-induced technique was used for the deposition of graphene layers onto surgical masks with superhydrophobic properties and improved protection against COVID-19 and can be reusable and recyclable [223]. Copper nanoparticles embedded in superhydrophobic coatings provided antibacterial properties [197,224]. Different kinds of nanocomposites were also applied for the preparation of hydrophobic and superhydrophobic surfaces. Their use enabled the development of repellent surfaces with antiviral and antibacterial properties and helped to stop pandemics [225].

The segmented polyimide-siloxane fibers, obtained by the electrospinning method, exhibited stable superhydrophobic properties, which were confirmed by the high value of the WCA of 167° [226].

Waterborne polyurethane-urea (PUU) dispersions (WPUD), synthesized from biobased polyester polyol and isophorone diisocyanate (IPDI), were modified with a trans-cyclohexanediol isobutyl silsesquioxane derivative (POSS-OH), which was grafted onto the PUU backbone. They were applied as a new finishing agent for textile materials. Selected polyester (PES) fabrics were coated by the WPUD with the addition of trans-cyclohexanediol isobutyl POSS, which was covalently bound to the polymeric chain (as was confirmed by the FTIR and NMR spectra). The prepared polyurethane-urea coatings showed good hydrophobic, mechanical, and thermal properties. The WCA ~140° was observed, and the water column values were higher than 30 cm for all coated fabrics. They are a more sustainable alternative than waterproof fluoropolymer coatings based on PTFE. WPUD-POSS-OH multifunctional textile coatings showed increased hydrophobicity while maintaining thermal, mechanical, and water column properties, highly needed in protective workwear and technical textile materials [227].

## 6. Summary, Conclusions, and Future Perspectives

Many valuable modifications of the properties of natural and synthetic fibers and textile materials with functional silanes, siloxanes, and silicones, as well as their numerous practical applications in the textile industry and materials science have been described in this review. Among softeners, functional, reactive silanes and silicone additives undoubtedly play a dominant role in the market of auxiliary chemical substances for the textile industry. However, the final selection of the type of softening preparation always depends both on the expected technical and aesthetic qualities of the textile product but also on the economic aspects. Thus, the currently recommended commercial formulas of softening agents are appropriately optimized for a specific, usually narrow purpose, mixture of the basic chemical compound responsible for obtaining the softening effect and various types of conditioning additives, e.g., the preparation of stable emulsions in the technological process or highlighting specific additional features of the finish. Less expensive fatty acid esters (waxes and paraffins) are frequently used as additives to fatty amino acids, which allow to obtain an increased smoothness effect of a textile product. The addition of paraffins containing 24 to 32 carbon atoms in the polymeric backbone was also very profitable. Additives with a shorter carbon chain have a boiling point too low, and those with longer chains form stable emulsions of an *oil-in-water* type under elevated pressure conditions. Fatty acid esters (waxes), e.g., glycerin monoester of stearic acid and polyethylene derivatives functionalized with hydroxyl, epoxy, carboxyl, and aldehyde groups belong to a few of the most commonly used additives to commercial forms of softeners. Silicones are also used as additives to other kinds of softeners [4].

Several nanotechnological techniques were applied in textile processing and their functionalization. They included the formation of nanocoatings, including the use of plasma for finishing, and supporting enzymes on textiles. Although a minimum effect on the strength, feel, handle, or breathability of textiles was observed, some of these technologies were tested and validated at the lab scale, while most of them are still in the research stages. Advances and developments in these areas of finishing continue to unfold; thus, it is expected that they will be progressively applied in order to prepare smart, intelligent, and interactive clothing in the future [228].

The application area of silicone additives in textiles processing depends on their molecular structure [57].

1. For sewing thread lubricant for polyester yarn, non-aqueous-based permethyl PDMS silicone oils and hydroxy-modified PDMSs, both in the form of oil or emulsion, are used. The non-aqueous-based permethyl PDMS silicone oils and hydroxy-modified PDMSs are also used as non-yellowing softeners. The 100% PDMS oils are used in kiss roll machines, and PDMS emulsions are used in exhaust apparatus.
2. The primary additives used for the scouring and bleaching of the textile materials are wetting agents and detergents. As antifoaming agents in the formulation, either silicone emulsion or 100% ethoxylated and propoxylated PDMSs are used.
3. For fiber finishing, especially for man-made fibers such as polypropylene and polyester, which are inherently hydrophobic, a polyether-based silicone surfactant is used as the wetting additive.
4. Amine-substituted silicones and polyether-based aminosilicones are mainly used for softening applications to ensure hydrophilic properties. Over 95% of textiles are modified with silicones as softeners to make the substrate very smooth, soft, silky, elastomeric, and resilient.
5. Silicone has the inherent property of increasing the color strength of a colored substrate, especially a dispersion dye to black, red, and navy blue.

Depending on the type and concentration of the emulsifier and the nature of aminosilicone, a micro- or macro-emulsion was used as a durable, hydrophobic softener with a high degree of softness. However, owing to their exceptional performance and customization properties, and their low environmental impact, there is a steadily increasing use of silicones in the textile industry that can predict a bright future. As previously anticipated, they are still one of the most innovative classes of textile finishing additives. The benefits of using silicone auxiliaries and a wide range of their advantages for the modification of textiles and nonwovens result from their low concentrations. Thus, they are able to replace organic materials. Moreover, methyl silicones are inert and do not have a significant negative environmental impact. The advantages of the modifications of textiles and nonwovens with silicone agents based on PDMS led to numerous replacements of organic materials and include superior performance and minimal concerns for the environment [44,52].

From world market analyses, the future for the market of silicone softeners seems to be very optimistic [229]. Due to their ability to mix easily during the rinse cycle of washing machines, the most preferred type are the liquid silicone softeners for textiles. The above factors are expected to strengthen the global market for silicone textile softeners in the coming years. Although the cost of silicone textile softeners is relatively high, the current trend of using new and improved products for better technical performance and aesthetic quality of textiles, suggests that it is expected that the global demand for silicone textile softeners will increase in the near future [230].

New silicone compounds or mixtures thereof still have great potential to be developed to improve or impart functional properties to textiles, and new applications of silicone-treated textiles in therapeutic medicine are expected [77].

Newer, exciting developments in the textile industry have been introduced to give textiles novel and innovative functions, e.g., easy to clean or stain resistant, flame retardant, and anti-microbial and anti-fogging properties. In addition, textiles for electronics [conductive textiles based on carbon nanotubes (CNT), graphene and other nanomaterials, textiles for sensor functions, textiles for batteries and energy storage], catalysts immobilized on textiles, textiles as tissue engineering substrates and textiles for the separation of oil from water, are becoming more and more important thanks to innovative methods of textile finishing, e.g., through appropriate surface modification techniques and the use of biomimetic concepts found in nature [231].

The future development of the fabric softener market is likely to be influenced by the use of ultra-concentrates if accepted by consumers. The second area relates to the role that the multi-functionality of fabric softeners will play in the future, with new, more effective ingredients and greater potential for additional perceptible benefits to consumers. The latest trend is multifunctional preparations for softening fabrics. These new products not only soften clothes but also make ironing easier, reduce creasing in the dryer, and provide protection against stains. Finally, manufacturers may also find new forms of delivery to facilitate the use of the softeners [232].

All over the world, over 900 different chemical compounds are used in the production of clothing, modifying the physicochemical and mechanical properties of textiles [1]. In the late 1990s, the annual sales of liquid fabric softeners in the United States were approximately USD 700 million (in supermarkets, drug stores, and mass stores) [232]. The world textile auxiliaries (including chemical finishing) market reached USD 17 billion in 2004, almost double the world dyes market. According to incomplete statistics, in 2004, the world production of textile auxiliaries exceeded 3.1 million tons, a total of nearly 100 categories, 15 thousand varieties, and annual consumption of 2.70–2.80 million tons [233]. The world production of auxiliary chemical substances for the textile industry in 2017 was estimated at 2.6 million tons with a value of approximately USD 7.8 billion [234]. The global market for fabric softeners and conditioners was valued at USD 17,545 million in 2018 and was expected to reach USD 23,529 million by 2025, posting a Cumulative Annual Growth Rate (CAGR) of 4.3% from 2018 to 2025 [235].

According to other analyses in 2020, the global textile auxiliaries market was valued at USD 9.52 billion and is expected to reach USD 12.70 billion by 202, at a CAGR of 4.2% during the forecast period [236,237].

## Data Availability

Not applicable.
